# Factors Controlling the Aluminum(I)‐*meta*‐Selective C−H Activation in Arenes

**DOI:** 10.1002/chem.202101944

**Published:** 2021-07-22

**Authors:** Jorge Juan Cabrera‐Trujillo, Israel Fernández

**Affiliations:** ^1^ Departmento de Química Orgánica I and Centro de Innovación en Química Avanzada (ORFEO-CINQA) Facultad de Ciencias Químicas Universidad Complutense de Madrid 28040 Madrid Spain

**Keywords:** activation strain, aluminum, bond activation, density functional theory calculations, selectivity

## Abstract

The so far poorly understood factors controlling the complete *meta*‐selectivity observed in the C−H activation reactions of alkylarenes promoted by aluminyl anions have been explored in detail by means of Density Functional Theory calculations. To this end, a combination of state‐of‐the‐art computational methods, namely the activation strain model of reactivity and energy decomposition analysis, has been applied to quantitatively unveil the origin of the selectivity of the transformation as well as the influence of the associated potassium cation. It is found that the selectivity takes place during the initial nucleophilic addition step where the key LP(Al)→π*(C=C) molecular orbital interaction is more stabilizing for the *meta*‐pathway, which results in a stronger interaction between the reactants along the entire transformation.

## Graphical abstract

**The factors controlling** the so‐far poorly understood complete *meta*‐selectivity observed in the C−H activation reactions of alkylarenes promoted by different aluminyl anions are revealed by state‐of‐the‐art computational methods.

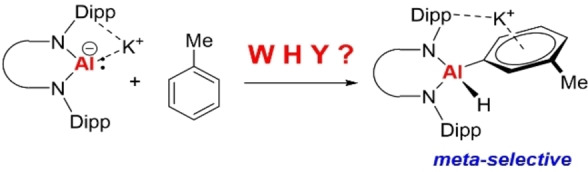

## Introduction

**Scheme 1:**
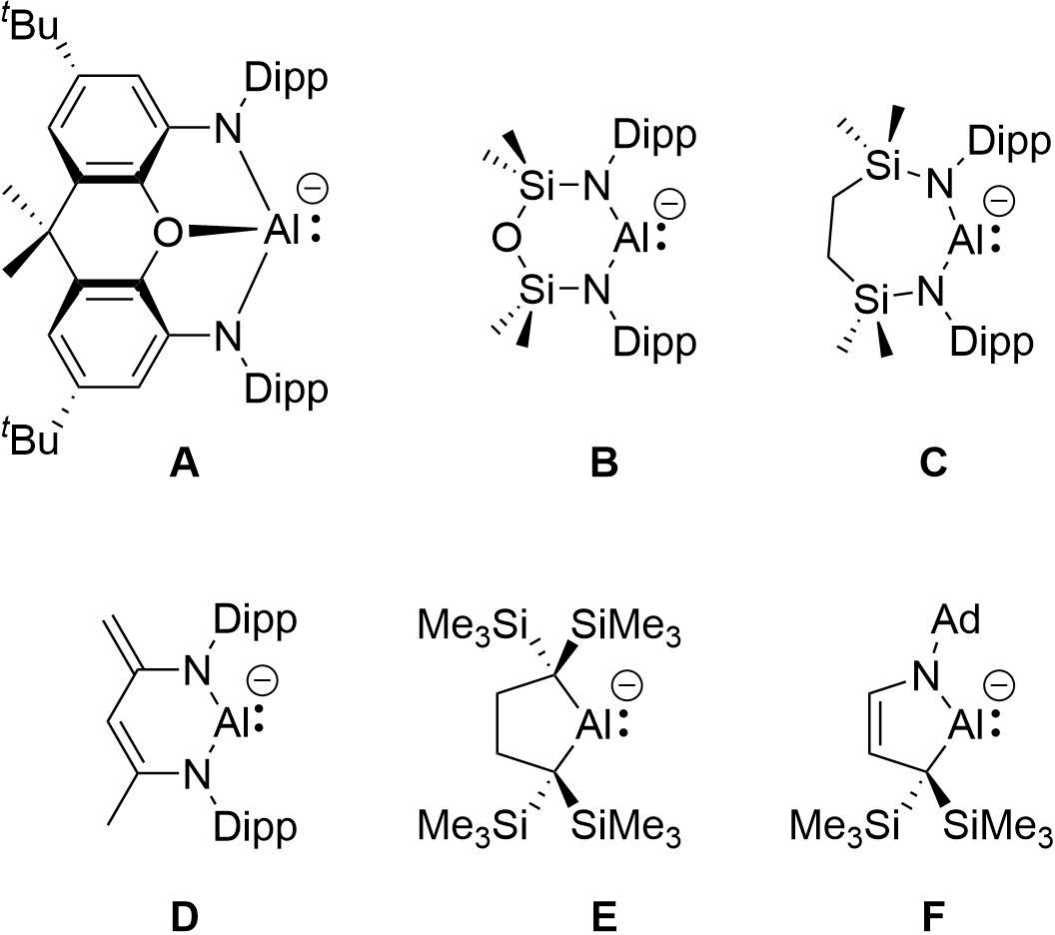
Most representative aluminyl anion species reported so far.

In contrast to neutral, low‐valent Al(I) compounds, which have been extensively studied since the isolation of [AlCp*_4_] in 1991,[^1^, ^2^] the chemistry of related anionic species, also known as aluminyl anions, is still in its infancy. Indeed, the discovery in 2018 of a new class of anionic Al(I) compound by Aldridge and co‐workers (**A** in Scheme 1)[^3^, ^4^] constituted the starting point of this new area of research in main group chemistry. Since then, other aluminyl species have been reported. For instance, the diamido supported systems reported by Coles (**B**),[^5^, ^6^] Hill and McMullin (**C**)^[7]^ and Harder (**D**)^[8]^ and the alkyl substituted species described by Yamashita (**E**)^[9]^ and Kinjo (**F**),^[10]^ should be particularly highlighted. Whereas the latter compounds are monomeric in the solid‐state, the diamido aluminyl anions were typically isolated as potassium dimeric species. To date, only **A** and **F** can be considered as truly ‘naked’ aluminyl anions as the corresponding Al⋅⋅⋅K interaction is practically negligible in the corresponding solid‐state.

**Scheme 2:**
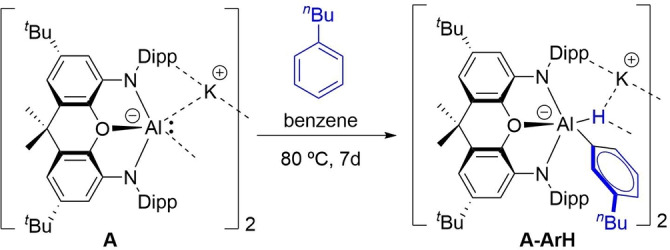
*meta*‐Selective C−H activation reported by Aldridge and co‐workers (see Ref. [12]).

The chemistry of this family of compounds is both rich and fascinating. Thus, a good number of different transformations including nucleophilic substitution, oxidative addition, cycloaddition and facile C−H and C−F bond activation reactions have been reported.^[11]^ In this sense, Aldridge and co‐workers recently reported that the C−H activation reactions of *n*‐butylbenzene mediated by dimeric **A** affords exclusively the corresponding activation products at the arene *meta* position (Scheme 2).^[12]^ A similar result was found when activating toluene and the isomers of xylene, although in these reactions benzylic C−H activation products were also observed. The same *meta*‐selectivity was reported nearly simultaneously by Yamashita and co‐workers in the analogous reactions involving **E**.^[13]^ Related nucleophilic^[14]^ and *meta*‐selective C−H activations involving transition metal complexes were also reported.^[15]^

By means of Density Functional Theory (DFT) calculations and using a simplified monomeric anionic model, it is proposed that the transformation proceeds through a hydride‐eliminating S_N_Ar reaction involving the nucleophilic attack of the electron‐rich Al(I) compound on the aromatic ring.^[12]^ Based on the computed charges on the corresponding Meisenheimer‐type transition state, the exclusive *meta*‐selectivity of this transformation was proposed to arise from the electron‐donating nature of the alkyl group in the arene which hampers the nucleophilic addition to the *ortho*‐ and *para*‐positions. A similar rationalization was used by Yamashita and co‐workers in the reactions involving **E**.^[13]^ Although this is indeed an elegant qualitative explanation, the physical factors governing the selectivity of this intriguing transformation are still poorly understood from a more quantitative point of view. For this reason, we decided to explore in detail those factors behind the observed *meta*‐selectivity as well as the influence of the potassium cation on the transformation. To this end, we will use a computational approach based on the combination of the Activation Strain Model (ASM)^[16]^ of reactivity and the Energy Decomposition Analysis (EDA)^[17]^ methods. This approach has been chosen because it has not only contributed to our current understanding of fundamental reactions in organic,^[18]^ organometallic,^[19]^ and main group^[20]^ chemistry but also because it has been particularly helpful to rationalize the experimentally reported C−C vs. C−H bond activation reactions in arenes (benzene, naphthalene and anthracene) promoted by aluminyl anion **A** ^[21]^ and related bond activation reactions mediated by neutral Al(I) species.^[22]^

## Computational Details

Geometry optimizations of the molecules were performed without symmetry constraints using the Gaussian09 (RevD.01)^[23]^ suite of programs at the dispersion corrected B3LYP^[24]^‐D3^[25]^/def2‐SVP^[26]^ level. Reactants and adducts were characterized by frequency calculations, and have positive definite Hessian matrices. Transition states (TS's) show only one negative eigenvalue in their diagonalized force constant matrices, and their associated eigenvectors were confirmed to correspond to the motion along the reaction coordinate under consideration using the Intrinsic Reaction Coordinate (IRC) method.^[27]^ Energy refinements were carried out by means of single‐point calculations at the accurate M06‐2X^[28]^‐D3 level using the much larger triple‐ζ basis set def2‐TZVPP^[26]^ and including solvent effects (solvent=toluene) with the Polarization Continuum Model (PCM) method.^[29]^ This level is denoted PCM(toluene)‐M06‐2X‐D3/def2‐TZVPP//B3LYP‐D3/def2‐SVP.

### Activation Strain Model of Reactivity and Energy Decomposition Analysis

Within the ASM method,^[16]^ also known as distortion/interaction model,[^16c^, ^16d^] the potential energy surface Δ*E*(ζ) is decomposed along the reaction coordinate, ζ, into two contributions, namely the strain Δ*E*_strain_(ζ) associated with the deformation (or distortion) required by the individual reactants during the process and the interaction Δ*E*_int_(ζ) between these increasingly deformed reactants [Eq. 1]:

(1) $$\Delta E\left(\zeta \right)=\Delta E{}_{\mathrm{strain}}\left(\zeta \right)+\Delta E{}_{\mathrm{int}}\left(\zeta \right)$$

Herein, the reaction coordinate is defined as the projection of the IRC onto the forming Al⋅⋅⋅C bond distance.

Within the EDA method,^[17]^ the interaction energy can be further decomposed into the following chemically meaningful terms [Eq. 2]:

(2) $$\Delta E{}_{\mathrm{int}}\left(\zeta \right)=\Delta V{}_{\mathrm{elstat}}\left(\zeta \right)+\Delta E{}_{\mathrm{Pauli}}\left(\zeta \right)+\Delta E{}_{\mathrm{orb}}\left(\zeta \right)$$

The term Δ*V*_elstat_ corresponds to the classical electrostatic interaction between the unperturbed charge distributions of the deformed reactants and is usually attractive. The Pauli repulsion Δ*E*_Pauli_ comprises the destabilizing interactions between occupied orbitals and is responsible for any steric repulsion. The orbital interaction Δ*E*_orb_ accounts for bond pair formation, charge transfer (interaction between occupied orbitals on one moiety with unoccupied orbitals on the other, including HOMO‐LUMO interactions), and polarization (empty‐occupied orbital mixing on one fragment due to the presence of another fragment). Moreover, the NOCV (Natural Orbital for Chemical Valence)^[30]^ extension of the EDA method has been also used to further partitioning the Δ*E*_orb_ term. The EDA‐NOCV approach provides pairwise energy contributions for each pair of interacting orbitals to the total bond energy.

The program package ADF^[31]^ was used for EDA calculations using the optimized B3LYP‐D3/def2‐SVP geometries at the M06‐2X level in conjunction with a triple‐ζ‐quality basis set using uncontracted Slater‐type orbitals (STOs) augmented by two sets of polarization functions with a frozen‐core approximation for the core electrons.^[32]^ Auxiliary sets of s, p, d, f, and g STOs were used to fit the molecular densities and to represent the Coulomb and exchange potentials accurately in each SCF cycle.^[33]^ Scalar relativistic effects were incorporated by applying the zeroth‐order regular approximation (ZORA).^[34]^ This level of theory is denoted ZORA‐M06‐2X/TZ2P//B3LYP‐D3/def2‐SVP.

## Results and Discussion

**Figure 1:**
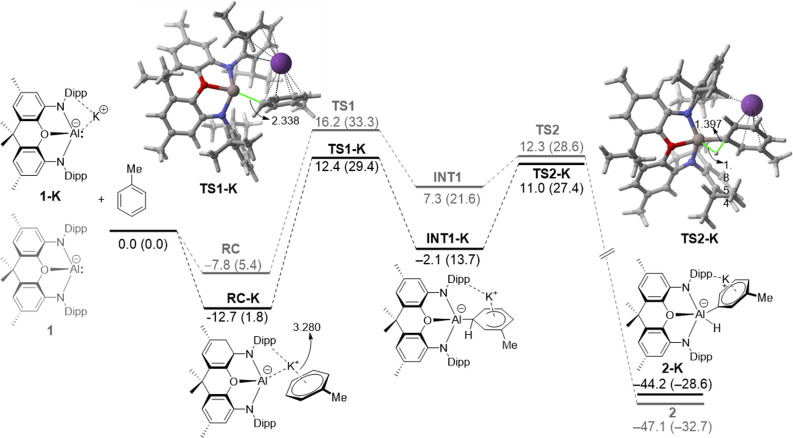
Computed reaction profiles for the *meta*‐C−H activation reactions involving the naked aluminyl anion **1** (grey lines) and its potassium counterpart **1‐K** (black lines). Relative energies (free energies at 298 K, Δ*G*, within parentheses) and bond distances are given in kcal/mol and angstroms, respectively. All data have been computed at the PCM(toluene)‐M06‐2X‐D3/def2‐TZVPP//B3LYP‐D3/def2‐SVP level.

We first explored the *meta*‐selective C−H activation reaction involving toluene and the monomeric aluminyl species **1**, described by Aldridge and co‐workers,^[12]^ where the bulky ^*t*^Bu groups in **A** were replaced by methyl groups. As depicted in Figure 1, the process begins with the exothermic formation of an initial reactant complex (**RC‐1**) where the toluene reactant weakly interacts with **1** (Δ*E*=−7.8 kcal/mol) mainly through dispersion and C−H⋅⋅⋅π noncovalent interactions. Not surprisingly, the formation of this species becomes endergonic (Δ*G*=5.4 kcal/mol) when thermal free energy corrections at 298.15 K are included, in accord with proper entropic corrections. From this species, the Meisenheimer intermediate **INT1** is formed in an endergonic reaction (Δ*G*_R_=21.6 kcal/mol from the separate reactants) via **TS1**, a saddle point associated with the formation of the new Al−C bond (Δ*G* ^≠^=33.3 kcal/mol). **INT1** evolves then into the final product **2** in a highly exergonic transformation (Δ*G*_R_=−32.7 kcal/mol from the separate reactants) via **TS2**, a saddle point associated with the migration of the key hydrogen atom from the aryl fragment to the aluminum atom (Δ*G* ^≠^=7.0 kcal/mol from **INT1**). The high exergonicity of the latter step compensates the previous endergonic S_N_Ar step and drives the transformation forward. Therefore, the transformation can be viewed as a stepwise reaction where the initial nucleophilic attack step is rate‐limiting.

**Figure 2:**
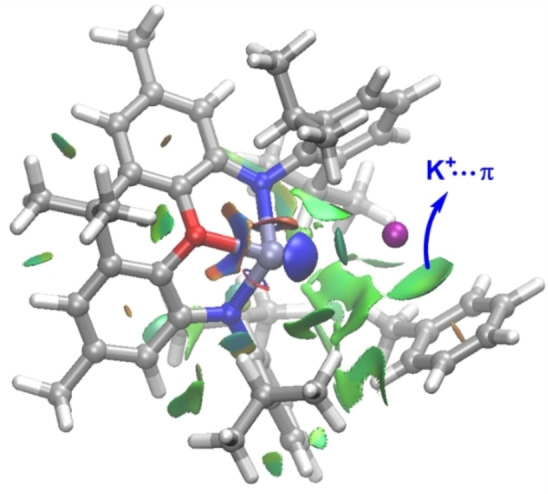
Contour plots of the reduced density gradient isosurfaces (density cutoff of 0.04 a.u.) for intermediate **RC‐K**. The green surfaces indicate attractive noncovalent interactions.

A rather similar stepwise mechanism was found for the analogous process involving **1‐K**, which includes the potassium cation in the calculations (Figure 1). The presence of potassium clearly favors the entire reaction pathway from the initial reactant complex **RC‐K** over the process involving the naked anion **1**.^[35]^ This is mainly due to the stabilizing interaction of the cation with the π‐system of toluene along the entire reaction coordinate and particularly, during the key S_N_Ar step. This cation‐π interaction can be easily visualized by means of the NCIPLOT method,^[36]^ which clearly confirms the occurrence of such stabilizing noncovalent interaction for the representative intermediate **RC‐K** (greenish surface, Figure 2). Two main consequences derive from this cation‐π interaction, (i) the reactants are in closer proximity than in the naked anionic system, and (ii) the toluene reactant is much more reactive towards the nucleophilic attack from the aluminyl center, i. e. the potassium cation acts as an electron‐withdrawing group for the S_N_Ar reaction. Indeed, the LUMO‐π* of toluene (*E*=−0.19 eV) becomes greatly stabilized upon coordination of potassium cation (*E*=−4.46 eV). Both effects result in a lower activation barrier for the process involving **1‐K** (ΔΔ*G* ^≠^=3.9 kcal/mol).^[37]^ Therefore, it becomes clear that, although the use of naked anions provides a similar qualitative information, the calculations involving these aluminyl systems should include the potassium cation in the corresponding reaction profiles. A similar finding was found by Harder and co‐workers in the related C−H activation of benzene promoted by **D**.^[8]^

**Figure 3:**
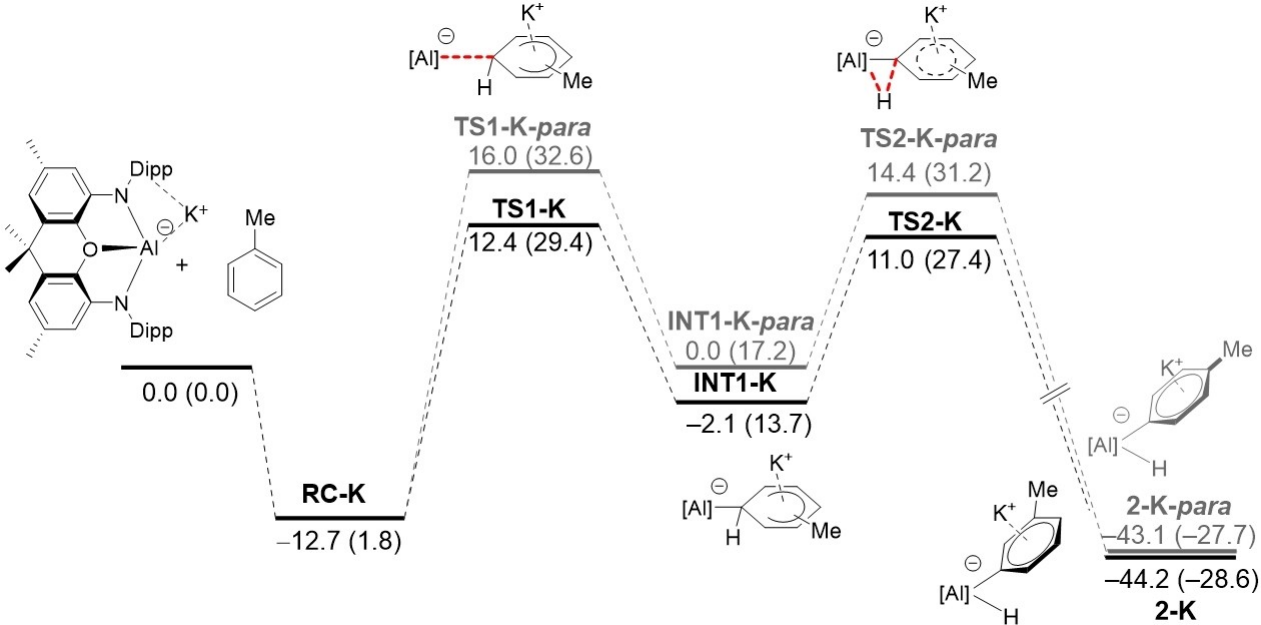
Computed reaction profiles for the *meta* (black lines) and *para* (grey lines) C−H activation reactions involving **1‐K** and toluene. Relative energies (free energies at 298 K, Δ*G*, within parentheses) are given in kcal/mol. All data have been computed at the PCM(toluene)‐M06‐2X‐D3/def2‐TZVPP//B3LYP‐D3/def2‐SVP level.

Once the mechanism involved in the C−H activation reaction in toluene promoted by **1‐K** has been explored, we then addressed the main aim of this work, namely, understanding the observed complete selectivity of the process leading to the exclusive formation of the corresponding *meta*‐reaction product. To this end, we compared the reaction profiles computed for the *meta‐* and *para*‐pathways involving **1‐K**. The *ortho*‐pathway can be safely ruled out as the corresponding transition states associated with the migration of the hydrogen atom to the aluminum center, **TS2‐K‐*ortho*** and **TS2‐K‐*ortho*’**, lie 4.1 and 4.0 kcal/mol, respectively, above the favored **TS2‐K**. This can be ascribed to unfavorable steric effects as suggested by Yamashita and co‐workers.^[13]^ From the data in Figure 3, it becomes evident that the *meta*‐pathway is energetically favored over the *para*‐counterpart along the entire reaction coordinate, from the initial transition state **TS1** up to the final reaction product. Interestingly, the key initial step involving the nucleophilic addition of the aluminyl anion to the K^+^‐activated toluene, where the selectivity of the transformation takes place, is both thermodynamically (ΔΔ*G*=3.5 kcal/mol) and kinetically favored for the *meta*‐pathway. Indeed, the computed barrier energy difference (ΔΔ*G* ^≠^=3.2 kcal/mol) is translated into a *meta/para* ratio of ca. 100 : 0 (at 298.15 K), which is fully consistent with the complete *meta*‐selectivity observed experimentally.^[12]^

To quantitatively understand the physical factors governing the selectivity of the transformation, the Activation Strain Model (ASM) of reactivity was applied next. To this end, we analyzed the evolution of the ASM terms from the common reactant complex **RC‐K** up to the corresponding transition states **TS1**. Although the ASM was originally developed for understanding bimolecular reactions, it can be also applied to unimolecular processes by careful selection of the interacting fragments.[^19c^, ^38^] According to the above‐commented effect of the potassium cation, we can safely select the naked anion **1** and the toluene‐K^+^ complex as fragments. Therefore, the barrier of the process arises then from the change in strain (ΔΔ*E*_strain_) and the change in interaction (ΔΔ*E*_int_) between these ionic fragments as one goes from the initial reactant complex **RC‐K** up to corresponding transition state structures.

**Figure 4:**
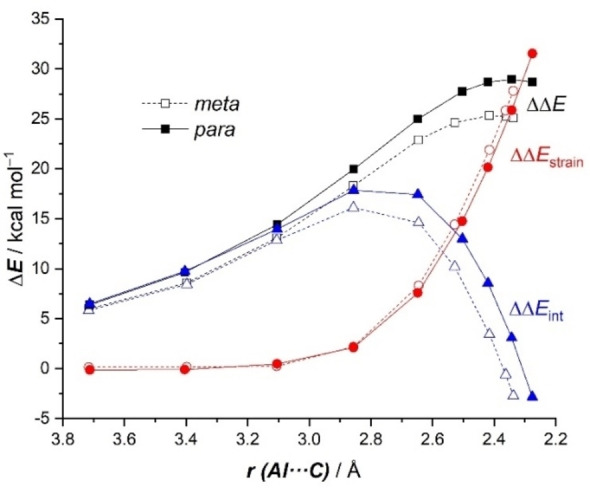
Comparative activation strain diagrams for the key *meta* (dotted lines) and *para* (solid lines) nucleophilic addition reaction involving **1‐K** and toluene along the reaction coordinate projected onto the forming Al⋅⋅⋅C bond distance. All data have been computed at the PCM(toluene)‐M06‐2X‐D3/def2‐TZVPP//B3LYP‐D3/def2‐SVP.

Figure 4 shows the corresponding Activation Strain Diagrams (ASDs) for both nucleophilic addition reactions. It becomes clear that the change in the strain energy (ΔΔ*E*_strain_), i. e. the energy penalty associated with the geometry distortion of both fragments, is nearly identical in both approaches, and therefore the ΔΔ*E*_strain_ term is not at all responsible for the barrier energy difference. At variance, the change in the interaction energy (ΔΔ*E*_int_), which only becomes stabilizing at the transition state region, is clearly more stabilizing for the *meta*‐pathway, practically along the entire reaction coordinate. Therefore, it can be concluded that the complete *meta*‐selectivity of the transformation mainly originates from the stronger interaction between the deformed reactants in the *meta*‐pathway as compared to the *para*‐pathway.

**Figure 5:**
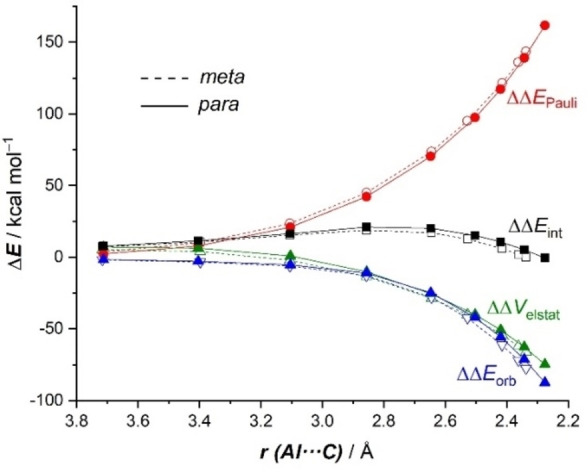
Comparative energy decomposition analyses for the key *meta* (dotted lines) and *para* (solid lines) nucleophilic addition reaction involving **1‐K** and toluene along the reaction coordinate projected onto the forming Al⋅⋅⋅C bond distance. All data have been computed at the ZORA‐M06‐2X/def2‐TZ2P//B3LYP‐D3/def2‐SVP level.

Our ASM analyses are therefore consistent with the qualitative arguments based on the computed charges reported by Aldridge and co‐workers^[12]^ and Yamashita and co‐workers,^[13]^ which suggest a preferential addition site in the aryl fragment (i. e. leading to a stronger interaction) due to the presence of the donor methyl group. To gain more quantitative insight into the reasons behind the computed stronger interaction in the *meta*‐pathway, we then applied the Energy Decomposition Analysis (EDA) method. Figure 5 graphically shows the evolution of the change of the EDA terms along the reaction coordinate, once again projected onto the Al⋅⋅⋅C bond‐forming distance, and using the aluminyl anion **1** and the cationic toluene‐K^+^ complex as fragments referenced to the common **RC‐K** intermediate. From the data in Figure 5, it becomes clear that although the *para*‐pathway benefits from a slightly less destabilizing Pauli repulsion (as a consequence of a less significant steric interaction between the fragments), the *meta*‐pathway benefits from both stronger electrostatic and orbital interactions along the entire reaction coordinate, and particularly, at the transition state region. For instance, at the same consistent Al⋅⋅⋅C bond‐forming distance of 2.4 Å, the difference in the changes of the electrostatic (ΔΔ*V*_elstat_) and orbital (ΔΔ*E*_orb_) terms are 2.5 and 4.7 kcal/mol, respectively. This indicates that the stronger interaction computed for the *meta*‐pathway, which is responsible for the lower barrier, derives mainly from more stabilizing orbital interactions between the deformed fragments as well as from stronger electrostatic attractions, albeit to a lesser extent.

**Figure 6:**
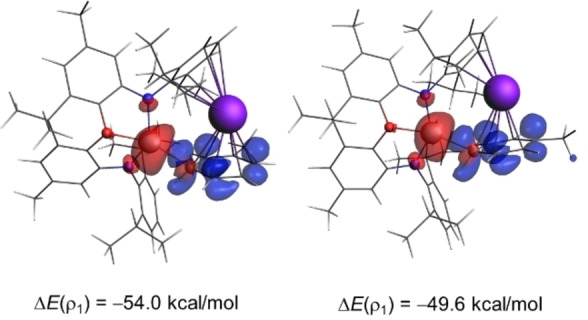
Plot of the deformation densities Δρ of the pairwise orbital interactions in the C−H activation reactions involving **1‐K** and toluene for the *meta* (left) and *para* (right) pathways, and associated stabilization energies Δ*E*(ρ_1_) computed at the same consistent Al⋅⋅⋅C bond‐forming distance of 2.4 Å. The color code of the charge flow is red→blue.

We then applied the Natural orbital for Chemical Valence (NOCV) extension of the EDA method, to not only identify but also quantify the main pairwise interactions responsible for the stronger orbital interactions computed for the *meta*‐pathway, which ultimately constitute the main factor behind the observed selectivity. The NOCV method locates a two‐electron donor‐acceptor interaction involving the donation from the lone‐pair of the aluminum atom to the π*(C=C) molecular orbital of the arene which dominates (>70 %) the total Δ*E*_orb_ term (see Figure 6 for the corresponding deformation densities of the reactions involving the *meta*‐ and *para*‐pathways at the same consistent Al⋅⋅⋅C bond‐forming distance of 2.4 Å).

**Figure 7:**
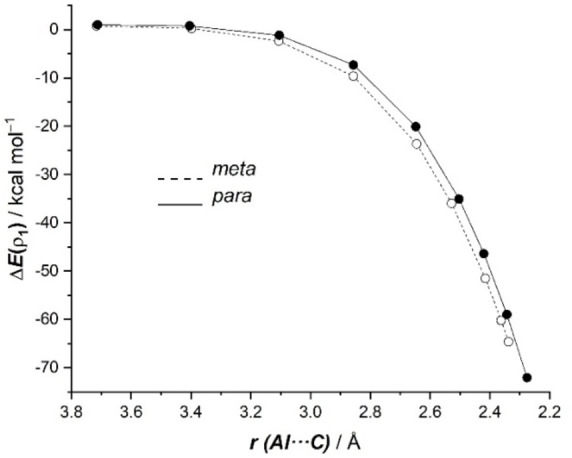
Evolution of the NOCV main contribution to the orbital interactions, Δ*E*(ρ_1_), for the key *meta* (dotted lines) and *para* (solid lines) nucleophilic addition reaction involving **1‐K** and toluene along the reaction coordinate projected onto the forming Al⋅⋅⋅C bond distance. All data have been computed at the ZORA‐M06‐2X/def2‐TZ2P//B3LYP‐D3/def2‐SVP level.

The evolution of this LP(Al)→π*(C=C) molecular orbital interaction, which corresponds to the HOMO‐LUMO interaction, along the reaction coordinate indicates that, in both approaches, this donor‐acceptor interaction continuously reinforces as the reaction progresses reaching its maximum at the respective transition states (Figure 7). This is exactly the same behavior as that found for the total Δ*E*_orb_ term (see Figure 5). Interestingly, this dominant LP(Al)→π*(C=C) interaction is markedly stronger for the *meta*‐pathway than for the *para*‐pathway. For instance, at the same consistent Al⋅⋅⋅C bond‐forming distance of 2.4 Å, the associated stabilization energy Δ*E*(ρ_1_) is −54.0 kcal/mol for the *meta*‐pathway whereas a lower value of −49.6 kcal/mol was computed for the *para*‐addition (see Figures 6 and 7). The corresponding energy difference ΔΔ*E*(ρ_1_)=4.4 kcal/mol roughly matches the difference in the total orbital attractions (ΔΔ*E*_orb_=4.7 kcal/mol, see above), which confirms the decisive role of this LP(Al)→π*(C=C) molecular orbital interaction in the selectivity of the transformation.

Our calculations therefore suggest that the *meta*‐selective C−H activation in alkylarenes promoted by the Aldridge‐Goicochea's aluminyl species **A** has mainly an orbital origin where the arene is activated by the potassium cation and the LP(Al)→π*(C=C) molecular orbital interaction is key. Together with stronger electrostatic attractions (although comparatively less decisive), both stabilizing interactions compensate the less destabilizing Pauli‐repulsion computed for the *para*‐pathway. This results in a stronger total interaction between the fragments which ultimately leads to the lower barrier computed for the *meta*‐pathway.

**Figure 8:**
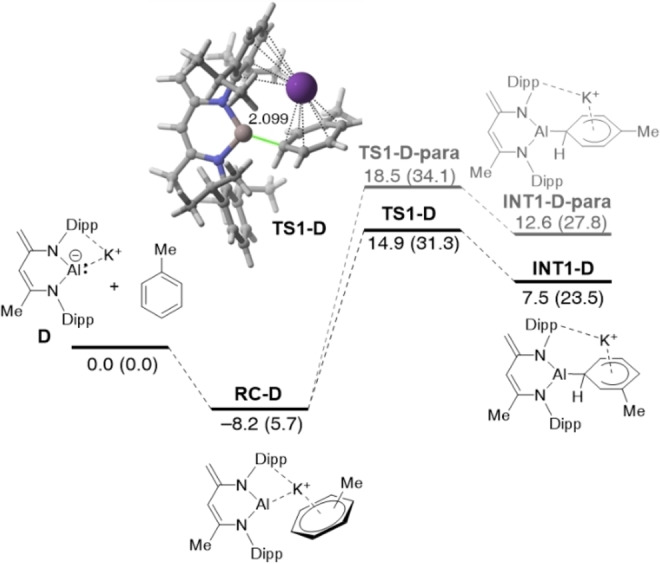
Computed reaction profiles for the key *meta* (black lines) and *para* (grey lines) nucleophilic addition reaction involving **D** and toluene. Relative energies (free energies at 298 K, ΔG, within parentheses) and bond distances are given in kcal/mol and angstroms, respectively. All data have been computed at the PCM(toluene)‐M06‐2X‐D3/def2‐TZVPP//B3LYP‐D3/def2‐SVP level.

To check the generality of this finding and the influence of the ligand attached to the Al(I) center on the process, we expanded our study to the analogous C−H activation reaction involving toluene and the Harder's aluminyl anion **D** (not experimentally reported so far). From the data in Figure 8, which shows the key initial nucleophilic addition (the complete reaction profile is given in Figure S2 in the Supporting Information), it is confirmed that the *meta*‐pathway is once again thermodynamically and kinetically favored over the *para*‐pathway. In comparison with the process involving **1‐K** (see above), while the activation barriers are only slightly higher, the corresponding Meisenheimer intermediates **INT1** are much less stabilized in the process promoted by **D** (by ca. 10 kcal/mol). This is mainly due to the stabilization provided by the oxygen atom in **INT1‐K** which is not present in **INT1‐D**. Indeed, the Second Order Perturbation Theory (SOPT) of the Natural Bond Orbital (NBO) method^[39]^ locates a strong delocalization of the oxygen lone‐pair into the p_z_ atomic orbital of the aluminum atom, LP(O)→p_z_(Al), in intermediate **INT1‐K** (associated SOPT energy Δ*E* ^(2)^=−23.5 kcal/mol and −33.7 kcal/mol, for the *meta*‐ and *para*‐Meisenheimer intermediates, respectively). On the other hand, the computed barrier energy difference (ΔΔ*G* ^≠^=2.8 kcal/mol) predicts an almost complete *meta*‐selective transformation (*meta/para* ratio of ca. 99 : 1, at 298.15 K).

A similar result was reported for the analogous process mediated by system **E**.^[13]^ To enable a direct comparison with our results, we re‐computed the reported corresponding profile at the PCM(toluene)‐M06‐2X‐D3/def2‐TZVPP//B3LYP‐D3/def2‐SVP level (see Figure S3 in the Supporting Information). Once again, it is found that the *meta*‐pathway is kinetically preferred (ΔΔ*G* ^≠^=2.5 kcal/mol) over the *para*‐pathway, which indicates that the *meta*‐selectivity in this transformation is general regardless of the ligands directly attached to the aluminum(I) center.

**Figure 9:**
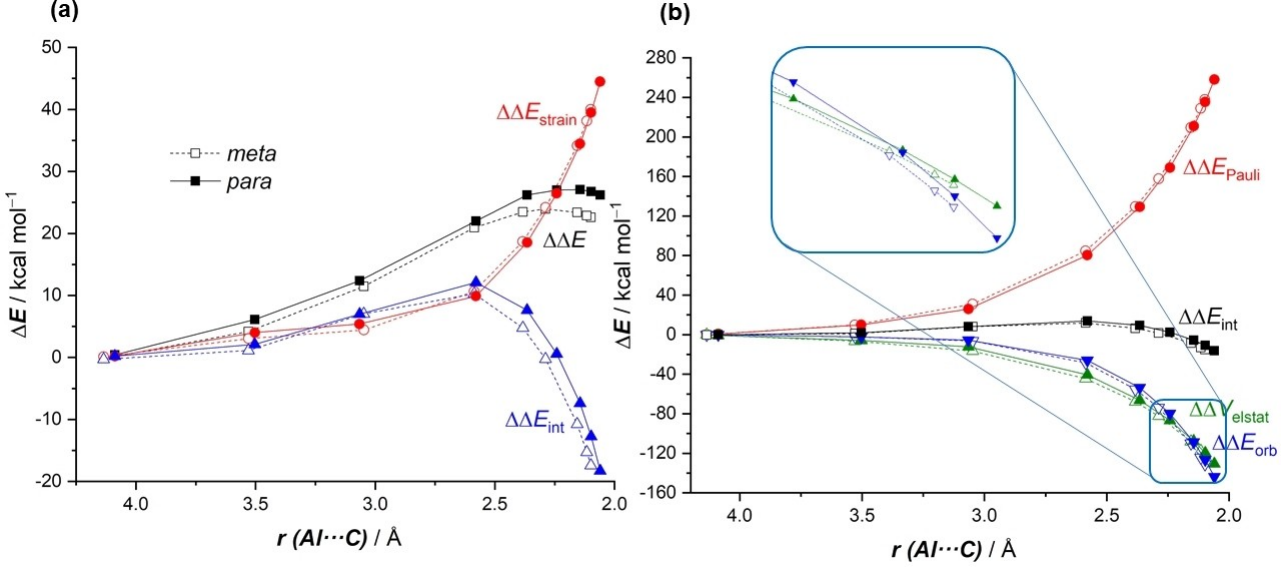
Comparative (a) activation strain diagrams (PCM(toluene)‐M06‐2X‐D3/def2‐TZVPP//B3LYP‐D3/def2‐SVP level) and (b) energy decomposition analyses (ZORA‐M06‐2X/def2‐TZ2P//B3LYP‐D3/def2‐SVP level) for the key *meta* (dotted lines) and *para* (solid lines) nucleophilic addition reaction involving **D** and toluene along the reaction coordinate projected onto the forming Al⋅⋅⋅C bond distance.

We applied next the ASM approach to unveil the factors behind the lower barrier computed for the *meta*‐pathway of the reaction involving toluene and Harder's aluminyl anion **D**. According to the ASDs in Figure 9a, it is confirmed that the change in the interaction energy is solely responsible for the *meta*‐selectivity as the ΔΔ*E*_int_ term becomes more stabilizing in the *meta*‐pathway from the initial reactant complex **RC‐D** up to the transition state **TS1‐D**. Once again, our calculations indicate that the change in the strain energy plays no role in the selectivity of the process as both approaches exhibit nearly identical ΔΔ*E*_strain_ values. The EDA method suggests that the stronger interaction in the *meta*‐pathway results from both more stabilizing orbital and electrostatic (albeit to a lesser extent) interactions (see Figure 9b), particularly at the transition state region. Once again, the key LP(Al)→π*(C=C) molecular orbital interaction dominates the orbital term in the process and is responsible for the higher orbital attractions in the *meta*‐pathway. For instance, at the same consistent Al⋅⋅⋅C bond‐forming distance of 2.20 Å, the corresponding stabilization energy in the *meta*‐pathway is −97.6 kcal/mol whereas a lower value of −91.9 kcal/mol was computed for the *para*‐pathway. Therefore, the *meta*‐selectivity (when alkyl groups are present in the arene) seems general in these aluminyl anion mediated C−H activations regardless of the nature of the ligand attached to the aluminum(I) center. In all cases, this selectivity originates almost exclusively from the stronger LP(Al)→π*(C=C) molecular orbital interaction computed for the *meta*‐pathway as compared to that in the alternative *para*‐pathway.

## Conclusion

From the computational study reported herein, the following conclusions can be drawn: (i) The C−H activation reaction of alkylarenes promoted by aluminyl species proceeds stepwise where the initial nucleophilic addition (SNAr‐like process) is rate‐limiting. (ii) The potassium cation should be included in the calculations involving these species as it leads to a stabilizing cation‐π noncovalent interaction which not only approximates the reactants but also activates the arene therefore reducing the barrier associated with the key S_N_Ar step. (iii) The complete *meta*‐selectivity of the transformation solely originates from a more stabilizing interaction between the reactants, which derives from stronger orbital and electrostatic (albeit to a lesser extent) attractions along the entire reaction coordinate, and particularly, at the transition state region, as compared to the alternative *para*‐pathway. (iv) According to the EDA‐NOCV method, the LP(Al)→π*(C=C) orbital interaction dominates the total orbital interactions in the process and is markedly stronger in the *meta*‐pathway. (v) This indicates that the *meta*‐selectivity in these C−H activation reactions has mainly an orbital control. (vi) Although these findings seem general and applicable to any aluminyl anion system, the ligands directly attached to the aluminum(I) center also play a role in the energetics of the process, and particularly in the stabilization of the key Meisenheimer intermediates.

## Conflict of interest

The authors declare no conflict of interest.

## Supporting information

As a service to our authors and readers, this journal provides supporting information supplied by the authors. Such materials are peer reviewed and may be re‐organized for online delivery, but are not copy‐edited or typeset. Technical support issues arising from supporting information (other than missing files) should be addressed to the authors.

Supporting Information
