# Supplementary material for: Factors Controlling the Aluminum(I)‐meta‐Selective C−H Activation in Arenes
Source: Chemistry. 2021 Jul 22;27(48):12422–9. doi: 10.1002/chem.202101944 (PMC8457071; doi:10.1002/chem.202101944)
Supplement: Supplementary file 1 — Supporting Information [file CHEM-27-12422-s001.pdf]

Chemistry–A European Journal

Supporting Information

Factors Controlling the Aluminum(I)-*meta*-Selective C–H Activation in Arenes

Jorge Juan Cabrera-Trujillo and Israel Fernández*

Contents:

1. Figure S1 and S2……………………………………………………………………S2

2. Figure S3……………………………………………………………………………S3

3. Cartesian coordinates and energies………………………………………………….....S4

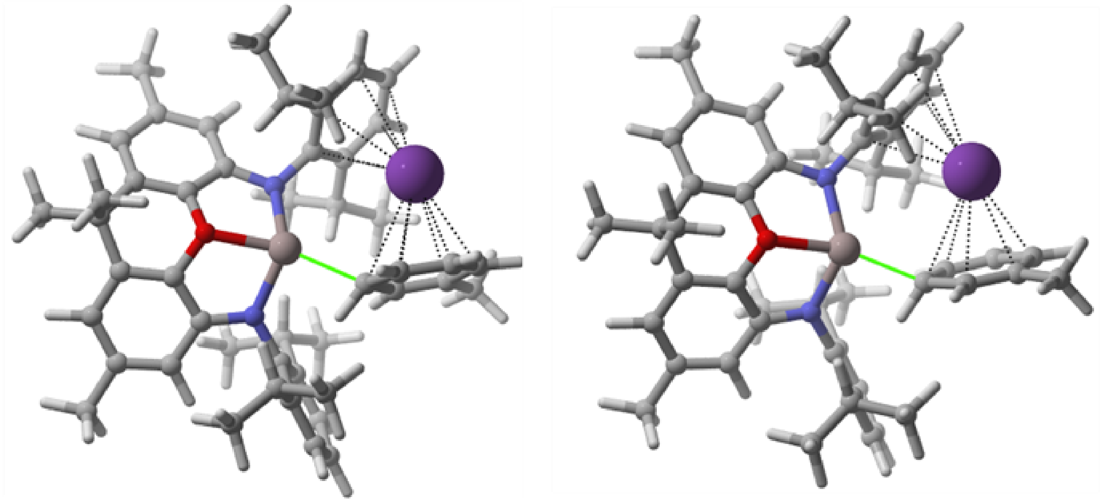

TS1-K
(E_{rel} = 0.0 kcal/mol)

TS1-K'
(E_{rel} = 0.4 kcal/mol)

Figure S1. Optimized transition states **TS1-K** and **TS1-K'** associated with the initial nucleophilic addition (meta-pathway). Energy values were calculated at the PCM(toluene)-M06-2X–D3/def2-TZVPP//B3LYP–D3/def2-SVP level.

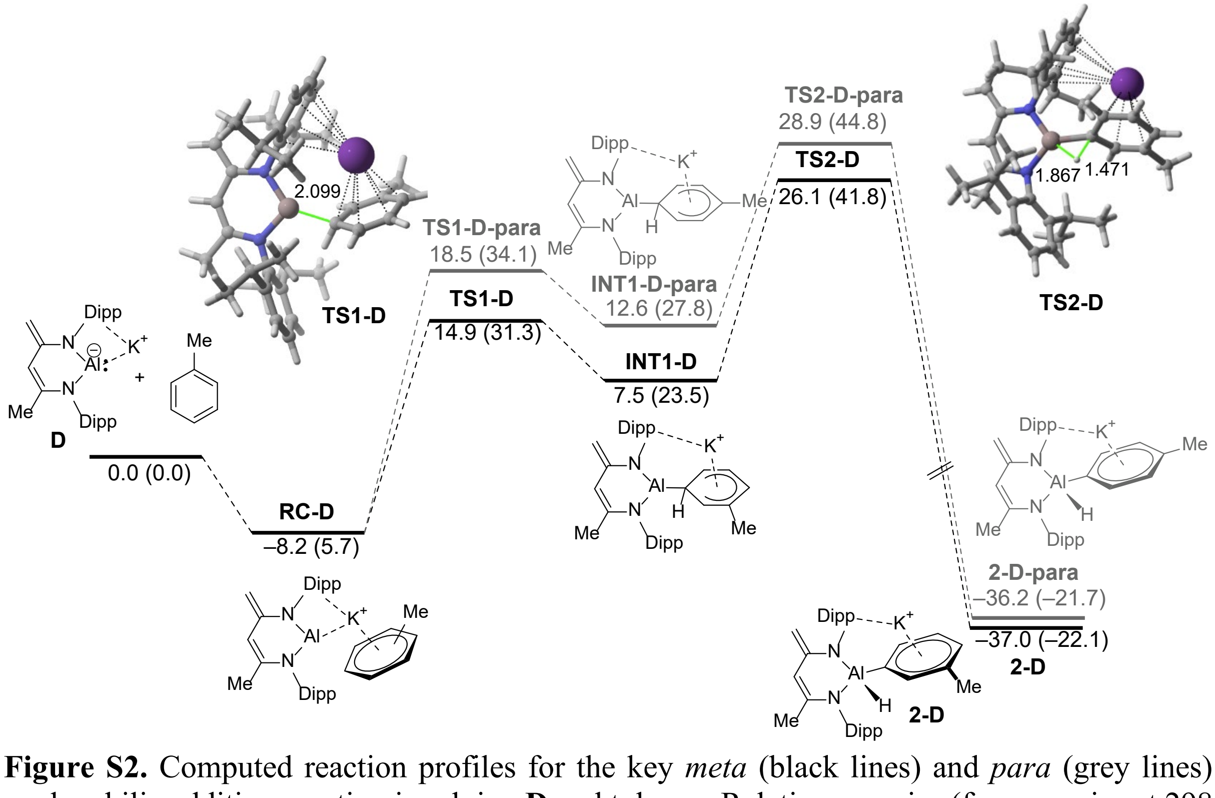

Figure S2. Computed reaction profiles for the key *meta* (black lines) and *para* (grey lines) nucleophilic addition reaction involving **D** and toluene. Relative energies (free energies at 298 K, ΔG, within parentheses) and bond distances are given in kcal/mol and angstroms, respectively. All data have been computed at the PCM(toluene)-M06-2X–D3/def2-TZVPP//B3LYP–D3/def2-SVP level.

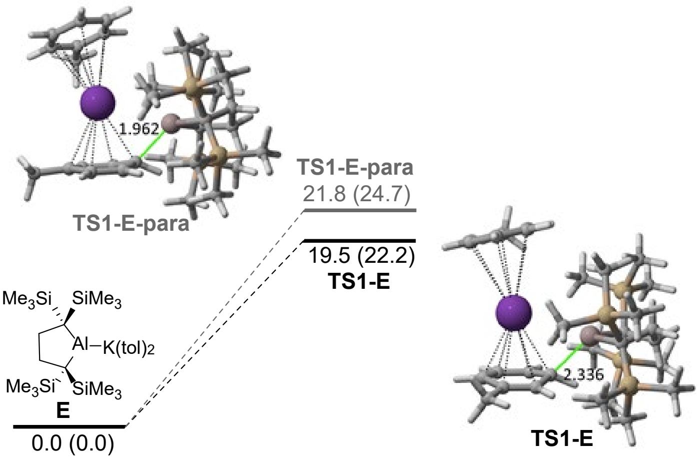

Figure S3. Computed reaction profiles for the key *meta* (black lines) and *para* (grey lines) nucleophilic addition reaction involving **E** and toluene. Relative energies (free energies at 298 K, ΔG, within parentheses) and bond distances are given in kcal/mol and angstroms, respectively. All data have been computed at the PCM(toluene)-M06-2X–D3/def2-TZVPP//B3LYP–D3/def2-SVP level.

Cartesian coordinates (in Å) and electronic ZPE corrected energies (in a.u) of all the stationary points discussed in the text. All geometry optimizations have been performed at the B3LYP-D3/def2-SVP level.

1: E= -2017.755482

C	-3.757527000	-1.536240000	1.286343000
C	-2.845795000	-1.364229000	0.210230000
C	-2.946719000	-2.197091000	-0.938400000
C	-3.957493000	-3.166782000	-0.991317000
C	-4.862191000	-3.328234000	0.059324000
C	-4.754494000	-2.517406000	1.187373000
N	-1.820451000	-0.394027000	0.286586000
Al	0.005828000	-0.836963000	1.287280000
C	-1.989730000	-2.000320000	-2.109582000
C	-1.710495000	-3.287478000	-2.894578000
C	-3.675163000	-0.676905000	2.543037000
C	-3.371764000	-1.525020000	3.788270000
N	1.774559000	-0.517852000	0.175060000
C	2.098727000	0.737752000	-0.276267000
C	3.262970000	1.147528000	-0.968720000
C	3.422688000	2.471564000	-1.412130000
C	2.422497000	3.429080000	-1.180333000
C	1.258544000	3.076658000	-0.468872000
C	1.166946000	1.767599000	-0.027794000
O	0.044890000	1.354007000	0.672828000
C	-1.145250000	1.870846000	0.180370000
C	-1.207899000	3.190255000	-0.229945000
C	0.088703000	4.013539000	-0.105919000
C	-2.443222000	3.645465000	-0.734406000
C	-3.533223000	2.764698000	-0.819207000
C	-3.394190000	1.413767000	-0.457519000
C	-2.158258000	0.901282000	0.010231000
C	4.694185000	2.852128000	-2.138385000
C	0.256501000	4.455098000	1.372163000
C	0.058710000	5.259396000	-0.999424000
C	-4.877186000	3.256703000	-1.309879000
C	2.840293000	-1.439401000	0.309906000
C	3.824530000	-1.248749000	1.320974000
C	4.879260000	-2.164721000	1.433158000
C	4.969818000	-3.270770000	0.591254000
C	3.982241000	-3.479420000	-0.372080000
C	2.913440000	-2.585454000	-0.529339000
C	3.744332000	-0.091956000	2.313332000
C	4.962601000	0.840913000	2.216873000
C	1.854589000	-2.811419000	-1.604780000
C	2.053736000	-1.857664000	-2.796206000
C	-2.485030000	-0.895431000	-3.060916000
C	-4.939487000	0.177996000	2.733197000
C	1.768913000	-4.263179000	-2.091393000
C	3.535413000	-0.605414000	3.747765000
H	-4.234998000	0.729182000	-0.588310000
H	-2.561089000	4.678621000	-1.064561000
H	2.556674000	4.445259000	-1.554415000
H	4.039324000	0.408490000	-1.178528000
H	1.198463000	5.013106000	1.501703000
H	0.279714000	3.580759000	2.038646000
H	-0.584485000	5.099202000	1.677649000
H	-0.059133000	4.989977000	-2.059725000
H	0.987214000	5.839855000	-0.888966000
H	-0.772708000	5.921934000	-0.714936000
H	-4.046132000	-3.809358000	-1.870029000

H	-5.644842000	-4.090440000	-0.000721000
H	-5.457558000	-2.650981000	2.014986000
H	-2.828602000	0.010568000	2.413943000
H	-4.173391000	-2.257035000	3.988385000
H	-3.273775000	-0.882154000	4.680030000
H	-2.424594000	-2.070331000	3.656128000
H	-5.118227000	0.815210000	1.853402000
H	-4.835099000	0.835484000	3.613146000
H	-5.834381000	-0.448970000	2.890105000
H	-1.038133000	-1.647915000	-1.683238000
H	-1.396667000	-4.106971000	-2.230112000
H	-0.909815000	-3.115837000	-3.631122000
H	-2.596737000	-3.626966000	-3.457077000
H	-3.452584000	-1.178657000	-3.511387000
H	-1.761198000	-0.733242000	-3.877966000
H	-2.622440000	0.058353000	-2.534479000
H	4.053196000	-4.358185000	-1.015213000
H	5.797900000	-3.978486000	0.693261000
H	5.637405000	-2.014861000	2.207633000
H	2.857692000	0.503351000	2.062214000
H	5.896708000	0.319305000	2.488099000
H	4.849732000	1.698700000	2.901556000
H	5.073073000	1.235289000	1.195234000
H	2.620802000	-1.215780000	3.805553000
H	3.429138000	0.239611000	4.449931000
H	4.386160000	-1.220360000	4.088324000
H	0.883971000	-2.559270000	-1.148218000
H	2.049680000	-0.808248000	-2.474115000
H	1.250135000	-1.988770000	-3.540608000
H	3.017593000	-2.059693000	-3.294892000
H	2.675371000	-4.564909000	-2.643277000
H	0.918884000	-4.384142000	-2.778024000
H	1.632967000	-4.964901000	-1.253172000
H	4.916706000	2.147035000	-2.957136000
H	4.631875000	3.863293000	-2.570479000
H	5.563977000	2.833229000	-1.457667000
H	-4.808648000	4.264808000	-1.748372000
H	-5.298200000	2.582156000	-2.074481000
H	-5.611700000	3.301390000	-0.485947000

1-K: E= -2617.610590

Al	0.010010000	-0.680908000	0.802935000
O	-0.084363000	1.442954000	0.690902000
N	-1.819770000	-0.349886000	0.156020000
C	-1.339122000	1.950150000	0.341998000
N	1.710419000	-0.172285000	-0.173350000
C	-2.284727000	0.946552000	0.075644000
C	-3.573468000	1.407190000	-0.264175000
H	-4.365354000	0.681433000	-0.459434000
C	-3.828475000	2.782307000	-0.412482000
C	-2.799589000	3.723520000	-0.237882000
H	-3.010325000	4.780181000	-0.406214000
C	-1.509785000	3.308136000	0.141807000
C	-0.272922000	4.206150000	0.334194000
C	0.940931000	3.426209000	-0.208835000
C	2.042224000	3.962691000	-0.900887000
H	2.086491000	5.030781000	-1.117137000
C	3.095913000	3.135433000	-1.324739000
C	3.047693000	1.748456000	-1.096476000
H	3.856417000	1.107288000	-1.454431000
C	1.951641000	1.165114000	-0.431830000

C	0.970090000	2.061130000	0.020232000
C	-0.062447000	4.440627000	1.853583000
H	-0.929660000	4.967747000	2.282085000
H	0.841605000	5.046303000	2.025880000
H	0.055333000	3.485329000	2.385834000
C	-0.438094000	5.560067000	-0.366316000
H	-0.589507000	5.437674000	-1.449085000
H	0.449137000	6.190515000	-0.207136000
H	-1.298617000	6.107266000	0.045709000
C	-2.758838000	-1.405476000	-0.011281000
C	-3.562846000	-1.821610000	1.080784000
C	-4.482255000	-2.863216000	0.888584000
H	-5.111096000	-3.185179000	1.723160000
C	-4.610422000	-3.493090000	-0.347393000
H	-5.334429000	-4.301581000	-0.480967000
C	-3.808548000	-3.088328000	-1.414931000
H	-3.917785000	-3.589526000	-2.378857000
C	-2.877632000	-2.049318000	-1.271913000
C	-2.042733000	-1.589143000	-2.462915000
H	-1.110669000	-1.167072000	-2.057263000
C	-1.664270000	-2.729587000	-3.416268000
H	-1.230919000	-3.583624000	-2.873842000
H	-0.926178000	-2.378316000	-4.154670000
H	-2.534444000	-3.098057000	-3.983565000
C	-2.744372000	-0.456668000	-3.234968000
H	-3.704557000	-0.808940000	-3.648778000
H	-2.117348000	-0.111578000	-4.074145000
H	-2.949842000	0.406032000	-2.587167000
C	-3.458914000	-1.158737000	2.449582000
H	-2.655401000	-0.411766000	2.393990000
C	-3.058750000	-2.165734000	3.539833000
H	-3.823162000	-2.948938000	3.676198000
H	-2.926362000	-1.656504000	4.509089000
H	-2.107393000	-2.656753000	3.278949000
C	-4.753183000	-0.414518000	2.819339000
H	-5.004152000	0.338246000	2.056734000
H	-4.640958000	0.104788000	3.785619000
H	-5.606275000	-1.107870000	2.908864000
C	2.804037000	-1.060262000	-0.090798000
C	2.877223000	-2.176423000	-0.978410000
C	3.900451000	-3.122883000	-0.800851000
H	3.972282000	-3.978166000	-1.474042000
C	4.853643000	-2.985716000	0.212306000
H	5.650965000	-3.725810000	0.321465000
C	4.784516000	-1.890339000	1.073242000
H	5.531824000	-1.784350000	1.864502000
C	3.769975000	-0.926435000	0.952527000
C	3.722077000	0.212971000	1.969695000
H	2.843458000	0.830172000	1.748732000
C	4.958207000	1.121956000	1.858741000
H	5.884796000	0.578085000	2.107643000
H	4.873552000	1.974555000	2.551860000
H	5.059045000	1.523861000	0.839943000
C	3.541935000	-0.304115000	3.407483000
H	2.592933000	-0.856986000	3.508423000
H	3.503204000	0.537515000	4.118125000
H	4.369531000	-0.963012000	3.719276000
C	1.875547000	-2.309090000	-2.123215000
H	0.878386000	-2.101510000	-1.702401000
C	2.134296000	-1.246218000	-3.206693000
H	3.125034000	-1.396468000	-3.667597000

H	2.097962000	-0.231093000	-2.790623000
H	1.374151000	-1.315010000	-4.001731000
C	1.826088000	-3.706788000	-2.749934000
H	2.769885000	-3.963613000	-3.258139000
H	1.030624000	-3.751544000	-3.506457000
H	1.620138000	-4.489688000	-2.001080000
K	1.486018000	-3.432997000	1.999098000
C	4.299369000	3.726148000	-2.023070000
H	4.114692000	4.760104000	-2.351415000
H	5.177344000	3.742320000	-1.353461000
H	4.582465000	3.133957000	-2.908470000
C	-5.225890000	3.235358000	-0.769133000
H	-5.904270000	3.144868000	0.097204000
H	-5.244224000	4.285966000	-1.096431000
H	-5.653877000	2.619452000	-1.576754000

Toluene: E= -271.253654

H	-0.012054000	0.737920000	2.154608000
C	0.030568000	2.426493000	0.000000000
H	-0.462945000	2.844101000	0.891276000
H	-0.462945000	2.844101000	-0.891276000
H	1.071215000	2.796065000	0.000000000
C	-0.005485000	0.917122000	0.000000000
C	-0.008049000	0.195276000	-1.204474000
C	-0.008049000	-1.202510000	-1.207267000
C	-0.007319000	-1.907897000	0.000000000
C	-0.008049000	-1.202510000	1.207267000
C	-0.008049000	0.195276000	1.204474000
H	-0.012054000	0.737920000	-2.154608000
H	-0.012215000	-1.743348000	-2.157500000
H	-0.010195000	-3.000914000	0.000000000
H	-0.012215000	-1.743348000	2.157500000

D: E= -2079.582627

Al	0.097467000	-0.140891000	-0.578534000
N	1.597483000	-0.087141000	0.559900000
N	-1.222338000	-0.045379000	0.776217000
C	0.340974000	-0.038498000	2.657000000
H	0.432289000	-0.029959000	3.744173000
C	1.512922000	-0.061946000	1.952870000
C	2.883903000	-0.010898000	-0.062321000
C	-1.036456000	-0.012566000	2.169371000
C	-2.558123000	0.006221000	0.300558000
C	3.571127000	-1.197490000	-0.421561000
C	3.442109000	1.256949000	-0.368383000
C	2.817929000	-0.062854000	2.717644000
C	-2.078804000	0.043414000	3.053210000
C	3.005576000	-2.572185000	-0.080462000
H	2.158514000	-2.414684000	0.602895000
C	-3.276086000	-1.197152000	0.060626000
C	-3.153146000	1.261081000	-0.005988000
C	5.327326000	0.148677000	-1.440013000
H	6.273735000	0.210593000	-1.984020000
C	4.785357000	-1.094131000	-1.115468000
H	5.316293000	-2.003688000	-1.408038000
C	4.658782000	1.312864000	-1.062019000
H	5.091011000	2.284773000	-1.314146000
C	-2.705573000	-2.545916000	0.481305000
H	-1.757663000	-2.342657000	0.997263000
C	4.023706000	-3.463283000	0.648952000
H	4.879475000	-3.723183000	0.004573000

H	3.551827000	-4.408205000	0.964856000
H	4.422271000	-2.965514000	1.547061000
C	-4.407491000	1.284255000	-0.640274000
H	-4.864680000	2.245177000	-0.892678000
C	-2.390664000	-3.439992000	-0.729633000
H	-3.293592000	-3.647704000	-1.329879000
H	-1.978803000	-4.409404000	-0.404912000
H	-1.636417000	-2.966782000	-1.381512000
C	2.456828000	-3.271930000	-1.335763000
H	1.682899000	-2.656383000	-1.821994000
H	2.010899000	-4.247853000	-1.079289000
H	3.258197000	-3.448113000	-2.073055000
C	-4.528797000	-1.129031000	-0.574155000
H	-5.080969000	-2.051648000	-0.774282000
C	2.729344000	2.547579000	0.025148000
H	1.912688000	2.275685000	0.708770000
C	-5.089516000	0.099653000	-0.938201000
H	-6.069015000	0.135617000	-1.423405000
C	-2.448759000	2.566852000	0.342716000
H	-1.534314000	2.299021000	0.888373000
C	2.091997000	3.222655000	-1.201896000
H	2.861691000	3.518216000	-1.934582000
H	1.534472000	4.128272000	-0.908773000
H	1.392599000	2.538228000	-1.709060000
C	-2.024549000	3.344325000	-0.914351000
H	-1.320388000	2.752934000	-1.524130000
H	-1.510231000	4.280314000	-0.641824000
H	-2.893150000	3.612551000	-1.540813000
C	-3.630089000	-3.256301000	1.483324000
H	-3.815200000	-2.613957000	2.357237000
H	-3.164249000	-4.189696000	1.839286000
H	-4.601924000	-3.520517000	1.033087000
C	-3.304551000	3.431718000	1.281956000
H	-4.237010000	3.769314000	0.798910000
H	-2.745871000	4.330436000	1.590281000
H	-3.568428000	2.867385000	2.189005000
C	3.646656000	3.523631000	0.778439000
H	4.109032000	3.043733000	1.655423000
H	3.072427000	4.395790000	1.131798000
H	4.459681000	3.903702000	0.138246000
H	2.641430000	-0.067858000	3.801420000
H	3.431688000	-0.940386000	2.458026000
H	3.428160000	0.819951000	2.467707000
K	-2.142684000	-0.121785000	-3.043839000
H	-1.884303000	0.068384000	4.126207000
H	-3.118758000	0.067066000	2.727048000

E: E= -3175.515226

Al	-0.745968000	0.044460000	0.075247000
C	-2.550263000	-0.717843000	0.748909000
Si	-2.306524000	-2.587026000	0.820479000
Si	-3.037181000	-0.016555000	2.439264000
C	-3.554986000	-0.323692000	-0.385753000
H	-3.564283000	-1.101594000	-1.169051000
H	-4.604639000	-0.275610000	-0.033062000
C	-3.930650000	-3.567602000	0.941579000
H	-4.433647000	-3.445898000	1.911723000
H	-3.738030000	-4.643759000	0.789672000
H	-4.632580000	-3.241930000	0.155653000
C	-1.498307000	-3.278540000	-0.766660000
H	-2.211001000	-3.279100000	-1.605135000

H	-1.197735000	-4.326884000	-0.597390000
H	-0.604323000	-2.715849000	-1.079938000
C	-1.168896000	-3.123943000	2.248341000
H	-0.195514000	-2.611656000	2.175187000
H	-0.993111000	-4.212318000	2.199489000
H	-1.593387000	-2.899988000	3.239188000
C	-3.975963000	1.628788000	2.303380000
H	-3.352993000	2.436021000	1.893960000
H	-4.307235000	1.937753000	3.309210000
H	-4.872822000	1.537062000	1.670080000
C	-4.219043000	-1.122181000	3.446417000
H	-5.133843000	-1.348019000	2.874429000
H	-4.520583000	-0.591322000	4.365669000
H	-3.766186000	-2.079487000	3.748127000
C	-1.528989000	0.315790000	3.550978000
H	-0.868394000	-0.561139000	3.630204000
H	-1.853346000	0.599244000	4.566838000
H	-0.929642000	1.145430000	3.141928000
C	-1.676815000	1.123152000	-1.434215000
Si	-1.045463000	2.890647000	-1.256075000
Si	-1.288904000	0.426472000	-3.150583000
C	-3.194391000	1.021538000	-1.062789000
H	-3.464023000	1.831953000	-0.363243000
H	-3.863313000	1.174424000	-1.933385000
C	-2.149732000	4.167994000	-2.127294000
H	-2.144135000	4.052301000	-3.221018000
H	-1.819089000	5.193757000	-1.889393000
H	-3.193168000	4.066427000	-1.784973000
C	-1.003972000	3.487899000	0.557706000
H	-2.020996000	3.588306000	0.966073000
H	-0.538153000	4.487757000	0.603764000
H	-0.436943000	2.820532000	1.227255000
C	0.732704000	3.094046000	-1.906666000
H	1.405708000	2.380366000	-1.406494000
H	1.099595000	4.114417000	-1.702240000
H	0.806590000	2.924475000	-2.991343000
C	-2.449626000	-0.989838000	-3.648598000
H	-2.311803000	-1.882974000	-3.025303000
H	-2.246866000	-1.273227000	-4.695268000
H	-3.507124000	-0.687855000	-3.583041000
C	-1.483694000	1.673024000	-4.581290000
H	-2.506493000	2.084080000	-4.601865000
H	-1.312647000	1.156436000	-5.541387000
H	-0.783880000	2.521274000	-4.529962000
C	0.487317000	-0.260579000	-3.283162000
H	1.243478000	0.502753000	-3.041584000
H	0.679240000	-0.620152000	-4.308830000
H	0.634731000	-1.105034000	-2.590013000
K	2.544831000	-0.312473000	0.590897000
C	2.277562000	-3.418849000	-1.258452000
C	3.527766000	-2.937877000	-1.692305000
C	4.581812000	-2.901890000	-0.761620000
H	5.564133000	-2.537927000	-1.076890000
C	4.396360000	-3.340611000	0.558084000
H	5.234617000	-3.316786000	1.259881000
C	3.146218000	-3.816828000	0.972227000
H	2.996626000	-4.158966000	1.999270000
C	2.084592000	-3.850194000	0.057490000
H	1.099010000	-4.197742000	0.372804000
C	1.758960000	2.946790000	3.373190000
H	1.200294000	3.868252000	3.154285000

H	2.103212000	2.984794000	4.417689000
H	1.041348000	2.112313000	3.290873000
C	2.906735000	2.753473000	2.415143000
C	4.070458000	2.063914000	2.804072000
H	4.159496000	1.700748000	3.832260000
C	5.118245000	1.844314000	1.899850000
H	6.017729000	1.316771000	2.228789000
C	5.022956000	2.316558000	0.583607000
H	5.844836000	2.159465000	-0.119612000
C	3.871213000	3.006796000	0.183740000
H	3.778573000	3.379673000	-0.839237000
C	2.825793000	3.219714000	1.089992000
H	1.927327000	3.745540000	0.761274000
C	3.713311000	-2.479001000	-3.117770000
H	3.626099000	-3.328152000	-3.816117000
H	2.937989000	-1.749868000	-3.402737000
H	4.699453000	-2.017022000	-3.273861000
H	1.440597000	-3.448534000	-1.960453000

RC: E= -2888.888841

K	2.763320000	-2.136494000	-0.457098000
C	4.634529000	0.877407000	-0.628524000
C	5.203619000	0.319818000	0.524223000
C	5.998850000	-0.827660000	0.417655000
C	5.679502000	-0.852588000	-2.006087000
C	4.876351000	0.296685000	-1.878063000
C	6.229661000	-1.411411000	-0.837251000
H	3.989752000	1.753082000	-0.547348000
H	5.013098000	0.782171000	1.494839000
H	4.421592000	0.738468000	-2.768960000
H	6.859370000	-2.303163000	-0.912421000
Al	0.057338000	-0.127451000	-0.433898000
O	-1.957256000	0.502411000	-0.828534000
N	-1.121176000	-1.680961000	0.203031000
N	0.002111000	1.728395000	0.285710000
C	-2.961554000	-0.428268000	-0.560175000
C	-2.477986000	-1.634015000	-0.022670000
C	-3.455735000	-2.616717000	0.236370000
H	-3.145475000	-3.586480000	0.631681000
C	-4.821605000	-2.343231000	0.042387000
C	-5.237796000	-1.076423000	-0.398814000
H	-6.304349000	-0.871304000	-0.497352000
C	-4.292641000	-0.079114000	-0.704970000
C	-4.594135000	1.356399000	-1.174715000
C	-3.554018000	2.276574000	-0.506967000
C	-3.777704000	3.574503000	-0.012731000
H	-4.771516000	4.020168000	-0.069203000
C	-2.728646000	4.310787000	0.562496000
C	-1.444423000	3.750322000	0.678998000
H	-0.641758000	4.321440000	1.150049000
C	-1.179375000	2.446598000	0.214938000
C	-2.262117000	1.792125000	-0.393453000
C	-4.393010000	1.425984000	-2.711916000
H	-5.097899000	0.749444000	-3.221085000
H	-4.563009000	2.452796000	-3.073174000
H	-3.370635000	1.128907000	-2.988222000
C	-6.028953000	1.777373000	-0.835527000
H	-6.213423000	1.742700000	0.248462000
H	-6.227098000	2.799618000	-1.189963000
H	-6.755423000	1.118523000	-1.333668000
C	-0.492316000	-2.913098000	0.470168000

C	-0.303072000	-3.875041000	-0.565067000
C	0.462463000	-5.023200000	-0.298890000
H	0.604905000	-5.766705000	-1.088126000
C	1.049220000	-5.231516000	0.950401000
H	1.644700000	-6.129158000	1.138045000
C	0.851350000	-4.293675000	1.968737000
H	1.295159000	-4.478493000	2.948459000
C	0.077399000	-3.140893000	1.758915000
C	-0.212611000	-2.166953000	2.896132000
H	-0.183097000	-1.154585000	2.466152000
C	0.801938000	-2.221546000	4.042373000
H	0.751601000	-3.175674000	4.592192000
H	1.835891000	-2.087878000	3.686057000
H	0.589004000	-1.422349000	4.768427000
C	-1.639713000	-2.374750000	3.437901000
H	-1.862497000	-1.638403000	4.227520000
H	-2.392461000	-2.259433000	2.647183000
H	-1.746628000	-3.383868000	3.870590000
C	-0.893238000	-3.683538000	-1.959834000
H	-1.419275000	-2.720680000	-1.969268000
C	0.194627000	-3.605568000	-3.044440000
H	0.804278000	-4.524058000	-3.086105000
H	-0.259386000	-3.464893000	-4.038914000
H	0.860070000	-2.744458000	-2.866996000
C	-1.925435000	-4.776027000	-2.288234000
H	-2.725146000	-4.801341000	-1.533507000
H	-2.392025000	-4.584514000	-3.268225000
H	-1.460112000	-5.775262000	-2.328530000
C	1.193459000	2.475852000	0.491702000
C	1.928619000	2.342511000	1.701564000
C	3.079157000	3.123451000	1.887530000
H	3.645645000	3.040800000	2.815825000
C	3.513127000	4.023586000	0.913808000
H	4.405335000	4.631451000	1.085070000
C	2.810875000	4.124694000	-0.284951000
H	3.167794000	4.808171000	-1.059893000
C	1.665386000	3.351775000	-0.525513000
C	0.986672000	3.451290000	-1.888768000
H	0.131522000	2.763370000	-1.890005000
C	0.441535000	4.861638000	-2.167304000
H	1.252445000	5.607760000	-2.211098000
H	-0.087507000	4.887658000	-3.134193000
H	-0.267545000	5.170307000	-1.385070000
C	1.931302000	2.985382000	-3.010520000
H	2.273355000	1.955180000	-2.826311000
H	1.414783000	3.003374000	-3.984567000
H	2.819505000	3.633960000	-3.091935000
C	1.450090000	1.387000000	2.790722000
H	1.133494000	0.464292000	2.276942000
C	0.218004000	1.940058000	3.528011000
H	0.476110000	2.865613000	4.069549000
H	-0.598577000	2.169409000	2.831212000
H	-0.155898000	1.207921000	4.263547000
C	2.540960000	0.996194000	3.794929000
H	2.160855000	0.238393000	4.493939000
H	3.428879000	0.578292000	3.293362000
H	2.869493000	1.855992000	4.401187000
C	-5.835908000	-3.429920000	0.316931000
H	-5.632022000	-3.939206000	1.273061000
H	-6.861128000	-3.031824000	0.356343000
H	-5.809872000	-4.203448000	-0.470607000

C	-2.962350000	5.720727000	1.055115000
H	-4.033727000	5.969596000	1.090619000
H	-2.545316000	5.868219000	2.064804000
H	-2.470795000	6.457002000	0.395314000
C	5.959451000	-1.447174000	-3.365603000
H	6.740121000	-0.869621000	-3.889916000
H	6.313710000	-2.486807000	-3.293760000
H	5.064030000	-1.430127000	-4.007172000
H	6.447313000	-1.269874000	1.311678000

RC-K: E= -2888.893051

K	2.668549000	-2.044718000	-1.536938000
C	5.247688000	0.445974000	-2.108700000
C	5.981387000	-0.736728000	-2.281194000
C	6.192255000	-1.581575000	-1.183559000
C	4.929874000	-0.062128000	0.258034000
C	4.725991000	0.778099000	-0.854198000
C	5.671399000	-1.243043000	0.074348000
H	5.073716000	1.113384000	-2.956367000
H	6.396128000	-0.990923000	-3.260127000
H	6.776112000	-2.498704000	-1.301001000
H	4.141796000	1.693005000	-0.729114000
Al	0.055041000	-0.087688000	-0.629881000
O	-2.031415000	0.313903000	-0.835259000
N	-0.845418000	-1.665906000	0.290707000
N	-0.121880000	1.790615000	0.005397000
C	-2.890345000	-0.618156000	-0.254690000
C	-2.226355000	-1.700430000	0.345378000
C	-3.060077000	-2.673639000	0.930021000
H	-2.607023000	-3.550020000	1.399322000
C	-4.457944000	-2.515936000	0.936461000
C	-5.048159000	-1.375454000	0.366534000
H	-6.132082000	-1.261515000	0.407638000
C	-4.255380000	-0.389275000	-0.249587000
C	-4.754325000	0.896866000	-0.938856000
C	-3.732506000	2.004959000	-0.619463000
C	-4.014881000	3.353967000	-0.333265000
H	-5.042933000	3.717403000	-0.351317000
C	-2.976656000	4.245063000	-0.013574000
C	-1.648405000	3.791945000	0.082255000
H	-0.859920000	4.480240000	0.393267000
C	-1.329296000	2.438405000	-0.154088000
C	-2.399823000	1.637338000	-0.585353000
C	-4.763102000	0.656245000	-2.471912000
H	-5.455718000	-0.161707000	-2.727144000
H	-5.082542000	1.569047000	-2.999505000
H	-3.760352000	0.384551000	-2.833209000
C	-6.167342000	1.276870000	-0.481138000
H	-6.204874000	1.456865000	0.603499000
H	-6.510308000	2.185927000	-0.996683000
H	-6.883113000	0.478618000	-0.727337000
C	-0.126545000	-2.877942000	0.341711000
C	-0.257350000	-3.846076000	-0.700844000
C	0.559971000	-4.988426000	-0.681346000
H	0.454460000	-5.735333000	-1.473161000
C	1.511999000	-5.185720000	0.318993000
H	2.140092000	-6.080595000	0.316943000
C	1.646436000	-4.233607000	1.333391000
H	2.385284000	-4.405862000	2.116891000
C	0.840144000	-3.084229000	1.372765000
C	0.964489000	-2.078363000	2.514988000

H	0.997167000	-1.076668000	2.055192000
C	2.237509000	-2.239681000	3.353233000
H	2.232052000	-3.180814000	3.927218000
H	3.146948000	-2.226044000	2.732698000
H	2.315198000	-1.417263000	4.078774000
C	-0.277561000	-2.116229000	3.424148000
H	-0.198298000	-1.354484000	4.216479000
H	-1.197278000	-1.921740000	2.858114000
H	-0.370538000	-3.102856000	3.908215000
C	-1.232209000	-3.669573000	-1.864498000
H	-1.744117000	-2.708654000	-1.739118000
C	-0.507830000	-3.602254000	-3.220290000
H	0.068415000	-4.518743000	-3.430957000
H	-1.233022000	-3.471771000	-4.039992000
H	0.175976000	-2.737390000	-3.254636000
C	-2.312609000	-4.764459000	-1.866547000
H	-2.855337000	-4.779833000	-0.910057000
H	-3.046558000	-4.582870000	-2.668464000
H	-1.876366000	-5.763981000	-2.030627000
C	1.007731000	2.573239000	0.364773000
C	1.439641000	2.610777000	1.718967000
C	2.564619000	3.380860000	2.047854000
H	2.905082000	3.428139000	3.083895000
C	3.266180000	4.093225000	1.074224000
H	4.142629000	4.684885000	1.352020000
C	2.840560000	4.048653000	-0.252100000
H	3.391454000	4.609179000	-1.012302000
C	1.713241000	3.302626000	-0.627961000
C	1.269627000	3.301682000	-2.087055000
H	0.346512000	2.710985000	-2.151287000
C	0.940219000	4.718787000	-2.584071000
H	1.831099000	5.368994000	-2.590333000
H	0.546553000	4.685115000	-3.613328000
H	0.178455000	5.191156000	-1.945098000
C	2.302936000	2.609056000	-2.990075000
H	2.441149000	1.563554000	-2.674155000
H	1.964380000	2.607185000	-4.039652000
H	3.280872000	3.119267000	-2.953811000
C	0.666653000	1.860379000	2.800383000
H	0.261436000	0.951545000	2.329531000
C	-0.539984000	2.677656000	3.298536000
H	-0.202048000	3.621531000	3.758976000
H	-1.228048000	2.927161000	2.480800000
H	-1.103951000	2.110375000	4.057656000
C	1.531567000	1.427345000	3.991485000
H	0.960578000	0.750444000	4.645996000
H	2.443295000	0.902555000	3.670831000
H	1.838181000	2.286896000	4.609650000
C	-5.315872000	-3.593228000	1.559732000
H	-4.949127000	-3.869294000	2.562037000
H	-6.364672000	-3.275268000	1.658471000
H	-5.302478000	-4.513043000	0.949122000
C	-3.273236000	5.704775000	0.244307000
H	-2.777866000	6.058227000	1.163490000
H	-2.903194000	6.336100000	-0.582292000
H	-4.352985000	5.891040000	0.347129000
C	4.311848000	0.285144000	1.585409000
H	4.720163000	-0.323683000	2.404587000
H	4.449628000	1.348984000	1.825440000
H	3.221132000	0.123374000	1.546958000
H	5.846213000	-1.903965000	0.928450000

RC-D: E= -2350.859402

K	1.411344000	2.620790000	-1.000043000
Al	0.180615000	-0.354426000	-0.293807000
N	-1.035670000	-1.741439000	0.109600000
N	1.745567000	-1.269206000	0.225942000
C	0.649074000	-3.383629000	0.767500000
C	-0.645813000	-3.014494000	0.525908000
C	-2.413223000	-1.370581000	0.093512000
C	1.876668000	-2.587898000	0.683268000
C	2.902694000	-0.449827000	0.146726000
C	-3.036685000	-0.928274000	1.290747000
C	-3.128251000	-1.336159000	-1.130764000
C	-1.747351000	-4.036864000	0.694153000
C	-2.382007000	2.775944000	-0.117645000
C	-2.459063000	2.667550000	-1.508994000
C	-1.895431000	3.652865000	-2.327457000
C	3.080017000	-3.134220000	1.033244000
C	-2.287901000	-0.957203000	2.620477000
C	3.252097000	0.391092000	1.238559000
C	3.630971000	-0.368150000	-1.070588000
C	-5.028895000	-0.334074000	0.020218000
C	-1.180989000	4.858073000	-0.343012000
C	-1.745878000	3.873294000	0.489233000
C	-4.337861000	-0.411342000	1.230717000
C	-1.252398000	4.751266000	-1.740290000
C	-4.425838000	-0.801869000	-1.146315000
C	2.546927000	0.266311000	2.583907000
C	-3.104632000	-1.602460000	3.750017000
C	4.625693000	0.614426000	-1.206882000
C	1.840388000	1.569561000	2.990407000
C	-1.819801000	0.454463000	3.009456000
C	4.260153000	1.354133000	1.062977000
C	-2.512957000	-1.865487000	-2.421411000
C	4.934359000	1.482145000	-0.155154000
C	3.333128000	-1.318004000	-2.224129000
C	-2.207317000	-0.730329000	-3.412894000
C	2.747938000	-0.578275000	-3.438567000
C	3.517246000	-0.215468000	3.675057000
C	4.572819000	-2.141523000	-2.608865000
C	-3.385650000	-2.951815000	-3.071093000
H	0.806025000	-4.420619000	1.069166000
H	-2.467404000	-3.728515000	1.469306000
H	-1.342588000	-5.019983000	0.969230000
H	-2.327720000	-4.142755000	-0.237551000
H	-2.819061000	1.986460000	0.498081000
H	-2.953940000	1.798963000	-1.947179000
H	-1.384932000	-1.567158000	2.480539000
H	-6.039285000	0.082183000	-0.010751000
H	-0.701348000	5.732539000	0.108455000
H	-4.818197000	-0.052902000	2.144913000
H	-4.976168000	-0.756980000	-2.089914000
H	1.773366000	-0.505136000	2.469318000
H	-3.993413000	-1.005248000	4.012571000
H	-2.491884000	-1.695923000	4.661431000
H	-3.450299000	-2.609600000	3.468625000
H	5.176600000	0.695051000	-2.148292000
H	2.552307000	2.401823000	3.122587000
H	1.299103000	1.441518000	3.941681000
H	1.099031000	1.864458000	2.229004000
H	-1.189050000	0.889046000	2.218314000

H	-1.231469000	0.436219000	3.941851000
H	-2.679443000	1.128177000	3.162117000
H	4.528892000	2.010201000	1.895948000
H	-1.553708000	-2.330382000	-2.150957000
H	5.718591000	2.235203000	-0.274243000
H	2.569916000	-2.022223000	-1.866469000
H	-3.129009000	-0.202503000	-3.712708000
H	-1.734385000	-1.124806000	-4.327816000
H	-1.521707000	0.005546000	-2.963124000
H	1.805615000	-0.070641000	-3.171178000
H	2.525300000	-1.282738000	-4.256806000
H	3.450729000	0.176504000	-3.832103000
H	3.967454000	-1.177409000	3.387915000
H	2.985029000	-0.358546000	4.629975000
H	4.328635000	0.511612000	3.848237000
H	5.385021000	-1.506532000	-3.001662000
H	4.318025000	-2.875960000	-3.390402000
H	4.956472000	-2.692328000	-1.736908000
H	-3.595491000	-3.770534000	-2.364773000
H	-2.877368000	-3.381727000	-3.949860000
H	-4.353857000	-2.550430000	-3.413043000
H	3.133964000	-4.166052000	1.383149000
H	4.010314000	-2.568063000	0.972835000
H	-0.829888000	5.541512000	-2.368062000
C	-1.646573000	3.973226000	1.991616000
H	-2.578092000	3.645103000	2.476575000
H	-0.845040000	3.321180000	2.378417000
H	-1.429779000	5.001424000	2.318497000
H	-1.962621000	3.569606000	-3.415258000

TS1: E= -2288.980429

Al	0.422587000	0.676933000	-0.900350000
O	-0.575034000	-1.153233000	-1.401572000
N	1.332000000	-0.579312000	0.320485000
N	-1.488347000	1.021619000	-0.331171000
C	-0.171020000	-2.202647000	-0.569544000
C	0.832288000	-1.866603000	0.358747000
C	1.198065000	-2.910183000	1.235139000
H	1.970115000	-2.718640000	1.983280000
C	0.575645000	-4.167423000	1.173451000
C	-0.444996000	-4.418315000	0.238606000
H	-0.929580000	-5.395764000	0.225077000
C	-0.840593000	-3.411315000	-0.659838000
C	-1.961037000	-3.493451000	-1.723323000
C	-2.737897000	-2.164471000	-1.611481000
C	-4.132915000	-2.016631000	-1.496200000
H	-4.796787000	-2.866610000	-1.659440000
C	-4.680749000	-0.778206000	-1.118687000
C	-3.843529000	0.296316000	-0.779251000
H	-4.272993000	1.203334000	-0.348707000
C	-2.438143000	0.177332000	-0.860493000
C	-1.970399000	-1.025241000	-1.431177000
C	-1.314006000	-3.608110000	-3.127619000
H	-0.715241000	-4.530700000	-3.196907000
H	-2.095036000	-3.634226000	-3.904851000
H	-0.654443000	-2.753282000	-3.331956000
C	-2.871668000	-4.704001000	-1.492327000
H	-3.347273000	-4.668882000	-0.500881000
H	-3.663537000	-4.744910000	-2.255692000
H	-2.296145000	-5.639071000	-1.568089000
C	2.615874000	-0.344118000	0.889714000

C	3.770814000	-0.965272000	0.327199000
C	5.029356000	-0.649607000	0.851505000
H	5.918310000	-1.109763000	0.410958000
C	5.180185000	0.252231000	1.905638000
H	6.175930000	0.495235000	2.287286000
C	4.047079000	0.836821000	2.465933000
H	4.159104000	1.537038000	3.298448000
C	2.761662000	0.547828000	1.986507000
C	1.563396000	1.198204000	2.668332000
H	0.660730000	0.844181000	2.154081000
C	1.566972000	2.732670000	2.569993000
H	2.416222000	3.167591000	3.124037000
H	1.642387000	3.071743000	1.529029000
H	0.634656000	3.141745000	2.990396000
C	1.457497000	0.765566000	4.141426000
H	0.523585000	1.145780000	4.586536000
H	1.463670000	-0.330471000	4.242627000
H	2.300895000	1.160571000	4.732566000
C	3.697261000	-1.958850000	-0.834203000
H	2.644116000	-2.089859000	-1.108561000
C	4.408958000	-1.453652000	-2.099876000
H	5.470126000	-1.221122000	-1.907860000
H	4.373247000	-2.227001000	-2.886880000
H	3.921159000	-0.548618000	-2.487538000
C	4.232620000	-3.341083000	-0.417287000
H	3.713048000	-3.719668000	0.475683000
H	4.086534000	-4.073209000	-1.229386000
H	5.312332000	-3.303595000	-0.193563000
C	-1.843921000	1.769433000	0.824289000
C	-2.117554000	1.096394000	2.054962000
C	-2.328865000	1.861872000	3.209252000
H	-2.524762000	1.350928000	4.156090000
C	-2.296194000	3.256087000	3.178657000
H	-2.450166000	3.832087000	4.095540000
C	-2.080395000	3.908009000	1.965418000
H	-2.085026000	4.999857000	1.938296000
C	-1.864765000	3.190007000	0.780467000
C	-1.747031000	3.921910000	-0.550514000
H	-1.187880000	3.269028000	-1.230389000
C	-3.140141000	4.115389000	-1.178092000
H	-3.784660000	4.730071000	-0.525929000
H	-3.059817000	4.620650000	-2.155651000
H	-3.639858000	3.149106000	-1.339635000
C	-1.002459000	5.260062000	-0.462615000
H	-0.029938000	5.149221000	0.039921000
H	-0.809898000	5.654337000	-1.472771000
H	-1.585063000	6.022702000	0.081782000
C	-2.255838000	-0.425336000	2.155393000
H	-2.026455000	-0.858866000	1.177000000
C	-3.707679000	-0.814145000	2.492803000
H	-3.979478000	-0.493899000	3.513299000
H	-4.417064000	-0.355456000	1.789678000
H	-3.835053000	-1.907991000	2.435512000
C	-1.283807000	-1.078352000	3.147207000
H	-1.463698000	-2.164882000	3.188647000
H	-0.245401000	-0.936012000	2.830018000
H	-1.394587000	-0.674361000	4.166754000
C	1.028093000	-5.255258000	2.121490000
H	2.027240000	-5.634129000	1.841802000
H	1.106568000	-4.880272000	3.155389000
H	0.335818000	-6.111550000	2.122694000

C	-6.179066000	-0.605066000	-1.017091000
H	-6.707374000	-1.569361000	-1.076942000
H	-6.462584000	-0.120939000	-0.067333000
H	-6.562967000	0.036125000	-1.829924000
C	3.083443000	1.947736000	-3.340714000
C	1.932748000	2.769320000	-3.589824000
C	1.186198000	3.201402000	-2.523230000
H	0.311689000	3.840038000	-2.679997000
C	1.445484000	2.606178000	-1.195187000
H	0.930887000	3.126030000	-0.372599000
C	2.884383000	2.401395000	-0.962846000
H	3.299276000	2.415751000	0.046127000
C	3.637842000	1.977481000	-2.034121000
H	3.688685000	1.599300000	-4.184145000
H	4.677238000	1.666687000	-1.877681000
C	1.602997000	3.167087000	-5.008858000
H	0.703250000	3.801790000	-5.059723000
H	2.435540000	3.713723000	-5.489428000
H	1.416320000	2.271673000	-5.630542000

TS1-K: E= -2888.846814

K	-3.607774000	-1.634044000	-1.366941000
C	-0.570286000	-2.432259000	-1.275446000
C	-1.127050000	-2.892996000	-2.561781000
C	-2.143493000	-3.824516000	-2.626940000
C	-2.175919000	-4.052241000	-0.200434000
C	-1.136054000	-3.139961000	-0.111254000
C	-2.753796000	-4.377407000	-1.465222000
H	0.522030000	-2.439493000	-1.266319000
H	-0.685846000	-2.510214000	-3.486556000
H	-2.488949000	-4.153162000	-3.614460000
H	-0.662103000	-2.985279000	0.861608000
H	-3.500341000	-5.170598000	-1.546600000
C	-2.669243000	-4.768592000	1.035689000
H	-3.745517000	-4.587205000	1.206505000
H	-2.549710000	-5.859737000	0.926238000
H	-2.125480000	-4.454429000	1.938556000
Al	-0.046107000	-0.223429000	-0.718301000
O	0.973645000	1.620520000	-0.833703000
N	-1.317358000	1.019251000	0.211112000
N	1.746719000	-0.708224000	-0.060029000
C	0.324545000	2.687379000	-0.210161000
C	-0.906526000	2.343532000	0.358064000
C	-1.588640000	3.391494000	1.006882000
H	-2.563872000	3.198076000	1.457725000
C	-1.020919000	4.673837000	1.101540000
C	0.249556000	4.928750000	0.557985000
H	0.686964000	5.921468000	0.670545000
C	0.956265000	3.917221000	-0.113151000
C	2.359302000	4.030675000	-0.738946000
C	3.081183000	2.711372000	-0.408787000
C	4.410014000	2.565843000	0.022615000
H	5.063398000	3.435420000	0.100916000
C	4.904585000	1.300502000	0.382802000
C	4.067777000	0.172545000	0.354406000
H	4.447960000	-0.791437000	0.695089000
C	2.723629000	0.276414000	-0.060893000
C	2.329834000	1.550400000	-0.487571000
C	2.208318000	4.153704000	-2.278171000
H	1.654037000	5.070291000	-2.535602000
H	3.199936000	4.193974000	-2.755827000

H	1.663530000	3.293322000	-2.693216000
C	3.123780000	5.248792000	-0.209576000
H	3.248656000	5.204404000	0.882471000
H	4.120458000	5.309557000	-0.670761000
H	2.594472000	6.179407000	-0.462543000
C	-2.720404000	0.835231000	0.292138000
C	-3.569911000	1.407320000	-0.703479000
C	-4.961284000	1.248766000	-0.587542000
H	-5.615738000	1.696205000	-1.340311000
C	-5.524111000	0.529685000	0.467981000
H	-6.609021000	0.423478000	0.548092000
C	-4.687683000	-0.053697000	1.424826000
H	-5.136607000	-0.615221000	2.244630000
C	-3.290819000	0.084770000	1.359824000
C	-2.395109000	-0.524403000	2.431636000
H	-1.521880000	-0.945216000	1.911299000
C	-3.055444000	-1.669425000	3.206871000
H	-3.873503000	-1.311337000	3.853297000
H	-3.459837000	-2.439344000	2.533121000
H	-2.317019000	-2.154828000	3.859874000
C	-1.877419000	0.559170000	3.394932000
H	-1.206852000	0.116537000	4.148183000
H	-1.319591000	1.340407000	2.862609000
H	-2.717482000	1.035615000	3.927200000
C	-3.018254000	2.173155000	-1.906246000
H	-1.932096000	2.254187000	-1.787667000
C	-3.255853000	1.409556000	-3.221427000
H	-4.328611000	1.228263000	-3.406346000
H	-2.866732000	1.982270000	-4.078467000
H	-2.722113000	0.443273000	-3.221665000
C	-3.573474000	3.604676000	-1.981758000
H	-3.378361000	4.148691000	-1.045756000
H	-3.091266000	4.159585000	-2.802443000
H	-4.660169000	3.616033000	-2.167341000
C	2.216502000	-2.016220000	0.286373000
C	2.014435000	-2.513181000	1.599873000
C	2.523632000	-3.778548000	1.928393000
H	2.375971000	-4.176167000	2.933467000
C	3.226187000	-4.540663000	0.997539000
H	3.619652000	-5.521780000	1.275771000
C	3.414324000	-4.048986000	-0.292939000
H	3.956451000	-4.653954000	-1.023818000
C	2.915316000	-2.797261000	-0.674281000
C	3.122820000	-2.322979000	-2.111009000
H	2.551240000	-1.391446000	-2.238230000
C	4.599914000	-1.998902000	-2.399332000
H	5.228882000	-2.897793000	-2.286142000
H	4.721342000	-1.633368000	-3.432494000
H	4.982738000	-1.225503000	-1.718693000
C	2.587111000	-3.338608000	-3.136598000
H	1.539631000	-3.609590000	-2.939120000
H	2.645699000	-2.919224000	-4.154662000
H	3.179444000	-4.267992000	-3.131572000
C	1.302426000	-1.672651000	2.656809000
H	0.559412000	-1.051123000	2.134422000
C	2.274085000	-0.702324000	3.353554000
H	3.061603000	-1.262077000	3.885477000
H	2.763787000	-0.032520000	2.634501000
H	1.739209000	-0.080137000	4.090625000
C	0.552898000	-2.511935000	3.699701000
H	-0.037123000	-1.857843000	4.359128000

H	-0.130118000	-3.235579000	3.228228000
H	1.244548000	-3.073914000	4.347182000
C	-1.797107000	5.774746000	1.786732000
H	-2.237226000	5.426569000	2.735126000
H	-1.162645000	6.646461000	2.006080000
H	-2.631959000	6.122799000	1.153421000
C	6.343638000	1.135085000	0.813571000
H	6.822465000	2.103786000	1.021536000
H	6.421907000	0.513695000	1.720322000
H	6.935083000	0.632604000	0.028445000

TS1-K-para: E= -2888.842282

K	-3.996058000	-0.510843000	-1.167966000
C	-1.293626000	-2.144440000	-0.992383000
C	-2.011314000	-2.527747000	-2.232338000
C	-3.253230000	-3.129899000	-2.224240000
C	-3.306852000	-3.096980000	0.180683000
C	-2.044832000	-2.527984000	0.224728000
C	-3.993125000	-3.393106000	-1.029183000
H	-0.271911000	-2.540570000	-0.977441000
H	-1.508973000	-2.360590000	-3.189805000
H	-3.692605000	-3.421437000	-3.186842000
H	-1.556671000	-2.418409000	1.195879000
Al	-0.097465000	-0.237479000	-0.657502000
O	1.407618000	1.231741000	-0.898150000
N	-0.913047000	1.351091000	0.245341000
N	1.506210000	-1.190060000	-0.037523000
C	1.122619000	2.457235000	-0.295819000
C	-0.129975000	2.503103000	0.324654000
C	-0.454161000	3.725145000	0.946177000
H	-1.425724000	3.839720000	1.430568000
C	0.462858000	4.790206000	0.967962000
C	1.729421000	4.648007000	0.376045000
H	2.438387000	5.474905000	0.431371000
C	2.085269000	3.454355000	-0.272911000
C	3.431905000	3.137562000	-0.951125000
C	3.756954000	1.675397000	-0.594076000
C	5.005500000	1.163836000	-0.203595000
H	5.884933000	1.808515000	-0.186034000
C	5.129394000	-0.179447000	0.191827000
C	4.001409000	-1.014995000	0.244172000
H	4.101629000	-2.035785000	0.615170000
C	2.726548000	-0.536995000	-0.125739000
C	2.699506000	0.780840000	-0.597510000
C	3.253832000	3.256412000	-2.488091000
H	2.976149000	4.286715000	-2.762097000
H	4.193012000	2.995232000	-3.000773000
H	2.466296000	2.579428000	-2.850106000
C	4.538736000	4.095670000	-0.498151000
H	4.695376000	4.047018000	0.589572000
H	5.488078000	3.851618000	-0.997125000
H	4.289837000	5.132488000	-0.768644000
C	-2.305112000	1.595832000	0.360083000
C	-2.978417000	2.349554000	-0.650072000
C	-4.346479000	2.630704000	-0.492419000
H	-4.863089000	3.221624000	-1.253466000
C	-5.059246000	2.170014000	0.615934000
H	-6.120703000	2.406162000	0.727065000
C	-4.403176000	1.405020000	1.585945000
H	-4.967516000	1.046797000	2.447798000
C	-3.034151000	1.107682000	1.481013000

C	-2.322965000	0.316647000	2.570615000
H	-1.573120000	-0.308099000	2.067221000
C	-3.248779000	-0.623224000	3.350150000
H	-3.961385000	-0.071955000	3.985483000
H	-3.820394000	-1.276574000	2.672540000
H	-2.656784000	-1.267468000	4.015126000
C	-1.560603000	1.261384000	3.517319000
H	-1.022796000	0.685691000	4.287153000
H	-0.823384000	1.866274000	2.971870000
H	-2.257456000	1.945515000	4.029669000
C	-2.265717000	2.854824000	-1.904989000
H	-1.206693000	2.584890000	-1.824807000
C	-2.790764000	2.165867000	-3.177629000
H	-3.872616000	2.328947000	-3.322387000
H	-2.278128000	2.559133000	-4.070020000
H	-2.589643000	1.080611000	-3.155529000
C	-2.337398000	4.385418000	-2.030092000
H	-1.936994000	4.869082000	-1.127181000
H	-1.740614000	4.727770000	-2.890712000
H	-3.371260000	4.737218000	-2.181656000
C	1.587582000	-2.559397000	0.373048000
C	1.314573000	-2.906448000	1.721323000
C	1.452976000	-4.246447000	2.113077000
H	1.247359000	-4.532359000	3.145849000
C	1.859208000	-5.226818000	1.210145000
H	1.967408000	-6.263916000	1.538119000
C	2.114439000	-4.879511000	-0.115614000
H	2.419395000	-5.653910000	-0.824028000
C	1.978260000	-3.558385000	-0.559276000
C	2.227821000	-3.238177000	-2.030797000
H	1.947492000	-2.186539000	-2.192197000
C	3.713658000	-3.381896000	-2.405288000
H	4.056954000	-4.419830000	-2.260110000
H	3.873252000	-3.121249000	-3.464701000
H	4.347374000	-2.723562000	-1.794254000
C	1.350991000	-4.096928000	-2.959792000
H	0.285798000	-4.031216000	-2.693327000
H	1.463436000	-3.765571000	-4.005491000
H	1.640467000	-5.159660000	-2.920328000
C	0.926649000	-1.839695000	2.741624000
H	0.373410000	-1.057106000	2.199718000
C	2.168182000	-1.159669000	3.347437000
H	2.783057000	-1.896406000	3.891276000
H	2.797390000	-0.700854000	2.573404000
H	1.870140000	-0.371112000	4.058588000
C	0.013141000	-2.366171000	3.855675000
H	-0.340884000	-1.532429000	4.480853000
H	-0.864218000	-2.893163000	3.449844000
H	0.543285000	-3.062167000	4.525064000
C	0.065032000	6.092375000	1.623921000
H	-0.438861000	5.919705000	2.588508000
H	0.935164000	6.740828000	1.805635000
H	-0.640943000	6.655246000	0.988135000
C	6.479705000	-0.742612000	0.570381000
H	7.208792000	0.053085000	0.786146000
H	6.409998000	-1.392562000	1.457268000
H	6.892912000	-1.358113000	-0.247873000
C	-5.278451000	-4.183937000	-1.048133000
H	-5.899015000	-3.948767000	-1.930736000
H	-5.106348000	-5.279836000	-1.073894000
H	-5.894401000	-3.986463000	-0.154240000

H	-3.781359000	-3.367520000	1.132203000

TS1-D: E= -2350.817853

K	2.581207000	1.181076000	-2.004518000
Al	-0.219094000	-0.190756000	-0.056981000
N	-1.801560000	-0.711095000	0.732508000
N	1.041610000	-1.100413000	0.934437000
C	-0.682744000	-2.250181000	2.269458000
C	-1.807737000	-1.682648000	1.741526000
C	-3.047616000	-0.225708000	0.212253000
C	0.727243000	-2.059331000	1.926439000
C	2.411985000	-0.993922000	0.555458000
C	-3.632810000	0.950087000	0.747667000
C	-3.659914000	-0.904534000	-0.872691000
C	-3.158736000	-2.141387000	2.237533000
C	-0.377564000	1.651770000	-1.049468000
C	-0.403615000	1.820291000	-2.539034000
C	0.356532000	2.766717000	-3.178935000
C	1.680892000	-2.825291000	2.530707000
C	-3.006034000	1.708534000	1.914171000
C	3.261663000	-0.051146000	1.196294000
C	2.920392000	-1.834530000	-0.474253000
C	-5.425589000	0.770972000	-0.893050000
C	1.144945000	3.687918000	-1.067716000
C	0.392776000	2.752609000	-0.379119000
C	-4.824003000	1.425127000	0.180145000
C	1.242018000	3.660771000	-2.494418000
C	-4.843417000	-0.384070000	-1.413932000
C	2.771729000	0.802064000	2.359171000
C	-3.874198000	1.614420000	3.181911000
C	4.254571000	-1.675166000	-0.885650000
C	3.110334000	2.289692000	2.181968000
C	-2.725496000	3.181674000	1.567862000
C	4.592536000	0.064897000	0.756904000
C	-3.031889000	-2.162325000	-1.467839000
C	5.087839000	-0.730780000	-0.279292000
C	2.050452000	-2.901671000	-1.131251000
C	-2.207248000	-1.818732000	-2.720992000
C	1.692771000	-2.524495000	-2.579686000
C	3.316804000	0.267221000	3.696499000
C	2.696169000	-4.294015000	-1.055276000
C	-4.057141000	-3.264841000	-1.771663000
H	-0.847391000	-2.993730000	3.050172000
H	-3.766488000	-1.296691000	2.594076000
H	-3.053279000	-2.867683000	3.053750000
H	-3.737503000	-2.610575000	1.425198000
H	-1.419184000	1.624095000	-0.661266000
H	-1.081977000	1.187136000	-3.117541000
H	-2.043344000	1.225809000	2.139837000
H	-6.349912000	1.162243000	-1.325888000
H	0.320125000	2.838496000	0.709892000
H	-5.287108000	2.328963000	0.583617000
H	-5.320932000	-0.888920000	-2.256705000
H	1.676999000	0.705388000	2.387793000
H	-4.859700000	2.082448000	3.022817000
H	-3.388308000	2.133762000	4.024092000
H	-4.045361000	0.570447000	3.483896000
H	4.652764000	-2.310653000	-1.680991000
H	4.195529000	2.475680000	2.237638000
H	2.639296000	2.885812000	2.979934000
H	2.741170000	2.672716000	1.220081000

H	-2.107536000	3.285569000	0.664378000
H	-2.193050000	3.674340000	2.397983000
H	-3.660299000	3.741143000	1.400687000
H	5.256164000	0.783304000	1.243747000
H	-2.334882000	-2.568975000	-0.720447000
H	6.127999000	-0.628726000	-0.600524000
H	1.112831000	-2.952766000	-0.561893000
H	-2.845729000	-1.378123000	-3.504196000
H	-1.720579000	-2.716937000	-3.135863000
H	-1.417413000	-1.083338000	-2.491897000
H	1.124966000	-1.578110000	-2.617715000
H	1.057158000	-3.296019000	-3.043306000
H	2.595573000	-2.416572000	-3.205339000
H	3.016659000	-0.778606000	3.852719000
H	2.928202000	0.866993000	4.535710000
H	4.418359000	0.323968000	3.723022000
H	3.624794000	-4.354036000	-1.646587000
H	2.004928000	-5.057045000	-1.448071000
H	2.932454000	-4.552759000	-0.012269000
H	-4.672541000	-3.493809000	-0.887377000
H	-3.543476000	-4.190085000	-2.079026000
H	-4.738199000	-2.983663000	-2.591024000
H	1.391854000	-3.558314000	3.284482000
H	2.740331000	-2.737940000	2.292429000
H	1.719899000	4.484486000	-3.029007000
C	1.861532000	4.786787000	-0.315218000
H	2.957507000	4.704980000	-0.427795000
H	1.578746000	5.775023000	-0.714746000
H	1.630764000	4.774063000	0.760015000
H	0.269448000	2.846496000	-4.269415000

TS1-D-para: E= -2350.813719

K	-2.800807000	1.535588000	1.464819000
Al	0.267271000	-0.217084000	0.095609000
N	1.890856000	-0.849650000	-0.490884000
N	-0.936022000	-1.370780000	-0.686709000
C	0.876498000	-2.788898000	-1.578889000
C	1.964382000	-2.053178000	-1.201866000
C	3.095625000	-0.174839000	-0.102421000
C	-0.551091000	-2.566642000	-1.339688000
C	-2.326378000	-1.197174000	-0.427960000
C	3.642236000	0.836903000	-0.931664000
C	3.702042000	-0.493955000	1.139769000
C	3.346614000	-2.564305000	-1.531999000
C	0.227620000	1.793260000	0.544531000
C	0.101492000	2.392706000	1.921409000
C	-0.691031000	3.482226000	2.191936000
C	-1.454222000	-3.517013000	-1.717059000
C	3.025651000	1.200367000	-2.279567000
C	-3.139789000	-0.478409000	-1.348051000
C	-2.893164000	-1.743545000	0.757561000
C	5.379879000	1.222022000	0.732626000
C	-1.389669000	3.640575000	-0.102570000
C	-0.601994000	2.559338000	-0.454984000
C	4.786409000	1.518841000	-0.492740000
C	-1.534237000	4.128149000	1.225618000
C	4.838319000	0.222186000	1.539952000
C	-2.570982000	0.065149000	-2.653036000
C	3.972421000	0.858500000	-3.444081000
C	-4.258939000	-1.534948000	1.017962000
C	-3.070396000	1.479326000	-2.985839000

C	2.604968000	2.679206000	-2.345690000
C	-4.502821000	-0.305563000	-1.049076000
C	3.115975000	-1.574783000	2.044729000
C	-5.062182000	-0.823544000	0.122569000
C	-2.053070000	-2.554271000	1.740258000
C	2.217913000	-0.952075000	3.128930000
C	-1.815305000	-1.782251000	3.050370000
C	-2.857493000	-0.903499000	-3.816472000
C	-2.658017000	-3.941554000	2.007033000
C	4.183944000	-2.482840000	2.672116000
H	1.090976000	-3.711236000	-2.119944000
H	3.922575000	-1.823306000	-2.106344000
H	3.295790000	-3.495050000	-2.111509000
H	3.926024000	-2.754370000	-0.613723000
H	1.302179000	1.790271000	0.239013000
H	0.715729000	1.969004000	2.721591000
H	-0.679686000	3.877028000	3.216487000
H	2.120399000	0.587662000	-2.402912000
H	6.267065000	1.770314000	1.059907000
H	-1.930977000	4.154596000	-0.907734000
H	-0.537267000	2.280377000	-1.510863000
H	5.217599000	2.301264000	-1.122112000
H	5.309176000	-0.003748000	2.499180000
H	-1.479436000	0.111989000	-2.528395000
H	4.900430000	1.451244000	-3.390723000
H	3.491322000	1.077233000	-4.411388000
H	4.256042000	-0.204838000	-3.441138000
H	-4.704179000	-1.951435000	1.925295000
H	-4.138935000	1.486782000	-3.257332000
H	-2.517331000	1.880237000	-3.850070000
H	-2.920697000	2.169538000	-2.142233000
H	1.902782000	2.946494000	-1.542786000
H	2.111701000	2.894508000	-3.307817000
H	3.476893000	3.348717000	-2.265609000
H	-5.140877000	0.235943000	-1.750740000
H	2.475498000	-2.215600000	1.420797000
H	-6.127025000	-0.685893000	0.329441000
H	-1.073565000	-2.719443000	1.271930000
H	2.794288000	-0.270229000	3.775515000
H	1.763046000	-1.730085000	3.764418000
H	1.398883000	-0.362774000	2.681034000
H	-1.275305000	-0.835918000	2.868383000
H	-1.197557000	-2.370459000	3.747932000
H	-2.765432000	-1.551439000	3.562411000
H	-2.442261000	-1.900122000	-3.611484000
H	-2.409605000	-0.524464000	-4.749647000
H	-3.944045000	-1.005432000	-3.979151000
H	-3.629500000	-3.880239000	2.524908000
H	-1.982312000	-4.536167000	2.642797000
H	-2.802705000	-4.485937000	1.061978000
H	4.849267000	-2.906979000	1.903626000
H	3.707274000	-3.318121000	3.210239000
H	4.812272000	-1.944083000	3.399736000
H	-1.111909000	-4.423871000	-2.216297000
H	-2.524984000	-3.406650000	-1.549779000
C	-2.226676000	5.434532000	1.530772000
H	-1.550361000	6.313226000	1.468516000
H	-3.054886000	5.629937000	0.827842000
H	-2.656576000	5.450450000	2.548503000

TS1-E: E= -3175.484748

Al	-0.591278000	0.068233000	0.088237000
C	-1.820056000	1.692722000	-0.167068000
Si	-2.604488000	2.498484000	1.370997000
Si	-0.843492000	3.028184000	-1.116859000
C	-2.949092000	1.044073000	-1.048849000
H	-3.749647000	0.645589000	-0.397630000
H	-3.456827000	1.786800000	-1.695677000
C	-4.191536000	3.430386000	0.889084000
H	-4.029872000	4.202786000	0.124503000
H	-4.625520000	3.916280000	1.779385000
H	-4.943541000	2.725172000	0.498339000
C	-3.192596000	1.314873000	2.736845000
H	-3.850536000	0.517410000	2.367161000
H	-3.777872000	1.915330000	3.454400000
H	-2.373144000	0.848144000	3.303473000
C	-1.456706000	3.708007000	2.289275000
H	-0.530103000	3.217236000	2.627670000
H	-1.984422000	4.067843000	3.188410000
H	-1.170591000	4.589457000	1.697648000
C	-0.504402000	2.606935000	-2.937044000
H	0.143692000	1.730740000	-3.065390000
H	0.002575000	3.470985000	-3.399951000
H	-1.435096000	2.431216000	-3.499975000
C	-1.721884000	4.712954000	-1.258158000
H	-2.690032000	4.602373000	-1.773894000
H	-1.096590000	5.382073000	-1.873568000
H	-1.906977000	5.216576000	-0.298556000
C	0.873217000	3.328431000	-0.349739000
H	0.836842000	3.528094000	0.730751000
H	1.365280000	4.184664000	-0.842138000
H	1.500439000	2.434884000	-0.504232000
C	-1.654179000	-1.215329000	-1.126107000
Si	-0.375000000	-2.094899000	-2.209683000
Si	-2.848625000	-2.495181000	-0.384024000
C	-2.444697000	-0.121292000	-1.934234000
H	-1.798543000	0.307466000	-2.716270000
H	-3.304381000	-0.541679000	-2.485832000
C	-1.135587000	-3.230615000	-3.533202000
H	-1.688185000	-4.083586000	-3.110806000
H	-0.347004000	-3.633958000	-4.191307000
H	-1.837032000	-2.659419000	-4.164413000
C	0.696983000	-0.890969000	-3.222439000
H	0.112444000	-0.344232000	-3.977951000
H	1.466123000	-1.475220000	-3.756321000
H	1.208543000	-0.155104000	-2.582537000
C	0.864306000	-3.125220000	-1.174041000
H	0.844800000	-2.840232000	-0.109372000
H	1.888526000	-2.986995000	-1.562095000
H	0.642022000	-4.202463000	-1.216036000
C	-3.683320000	-1.987560000	1.241640000
H	-2.975356000	-1.819944000	2.067717000
H	-4.374764000	-2.787864000	1.555495000
H	-4.277007000	-1.070731000	1.110043000
C	-4.287481000	-2.837581000	-1.580291000
H	-4.962715000	-1.970984000	-1.669172000
H	-4.884996000	-3.685248000	-1.204478000
H	-3.932324000	-3.093182000	-2.590748000
C	-2.072290000	-4.200445000	-0.032612000
H	-1.789091000	-4.734259000	-0.952138000
H	-2.832231000	-4.812913000	0.481212000
H	-1.184274000	-4.166075000	0.614585000

K	2.597751000	-0.642064000	0.907611000
C	2.056440000	-1.579454000	3.749401000
C	1.599630000	-0.240151000	3.874476000
H	2.141551000	0.449388000	4.529793000
C	0.477663000	0.207934000	3.202549000
H	0.149036000	1.243453000	3.310398000
C	-0.212511000	-0.662491000	2.274425000
H	-1.283646000	-0.439917000	2.109180000
C	0.101063000	-2.069429000	2.395146000
H	-0.545630000	-2.791776000	1.897661000
C	1.233237000	-2.516461000	3.062977000
C	4.095692000	1.325243000	-1.691416000
C	4.841404000	1.724400000	-0.564433000
C	5.707361000	0.783987000	0.022361000
H	6.298024000	1.071692000	0.896894000
C	5.833728000	-0.510800000	-0.501911000
H	6.520604000	-1.220723000	-0.033400000
C	5.092294000	-0.888668000	-1.628160000
C	4.221836000	0.036500000	-2.221066000
H	3.630735000	-0.249030000	-3.093552000
C	1.584233000	-3.985061000	3.089646000
H	1.618425000	-4.366948000	4.123649000
H	0.858644000	-4.589044000	2.525019000
H	2.583100000	-4.163935000	2.653659000
H	2.917098000	-1.926118000	4.326785000
H	3.407923000	2.035139000	-2.158701000
C	4.714089000	3.125890000	-0.020286000
H	5.248626000	3.243193000	0.933773000
H	5.132078000	3.858360000	-0.731122000
H	3.658592000	3.398624000	0.135347000
H	5.192979000	-1.894276000	-2.044035000

TS1-E-para: E= -3175.481083

Al	-0.606754000	0.001178000	0.065839000
C	-1.751226000	1.676737000	-0.244695000
Si	-2.348344000	2.729013000	1.231664000
Si	-0.790039000	2.803975000	-1.443727000
C	-2.999975000	1.012315000	-0.938043000
H	-3.760435000	0.763600000	-0.175103000
H	-3.514392000	1.707856000	-1.630054000
C	-3.961531000	3.625919000	0.772819000
H	-3.860792000	4.252255000	-0.125373000
H	-4.279775000	4.274435000	1.606574000
H	-4.773205000	2.903783000	0.586958000
C	-2.769889000	1.801082000	2.834330000
H	-3.485428000	0.981533000	2.693231000
H	-3.237004000	2.532288000	3.516290000
H	-1.883405000	1.401203000	3.350171000
C	-1.113074000	4.051541000	1.821434000
H	-0.154527000	3.611897000	2.139704000
H	-1.550749000	4.552407000	2.701241000
H	-0.893980000	4.827185000	1.073700000
C	-0.508922000	2.050963000	-3.164876000
H	0.100730000	1.138248000	-3.138650000
H	0.023411000	2.794547000	-3.782836000
H	-1.455584000	1.814255000	-3.676122000
C	-1.668974000	4.445035000	-1.844048000
H	-2.666760000	4.257241000	-2.274496000
H	-1.081408000	4.991492000	-2.601373000
H	-1.798571000	5.109857000	-0.977763000
C	0.958203000	3.210152000	-0.810867000

H	0.972699000	3.815312000	0.105430000
H	1.506245000	3.765276000	-1.591426000
H	1.508372000	2.276649000	-0.606819000
C	-1.884441000	-1.338734000	-0.828171000
Si	-0.817270000	-2.481664000	-1.894911000
Si	-3.077357000	-2.377078000	0.225756000
C	-2.670947000	-0.293634000	-1.701895000
H	-2.079511000	-0.024581000	-2.592197000
H	-3.610965000	-0.707877000	-2.109680000
C	-1.820542000	-3.710903000	-2.944092000
H	-2.394044000	-4.429667000	-2.338774000
H	-1.151051000	-4.287477000	-3.605038000
H	-2.536901000	-3.169474000	-3.584552000
C	0.246273000	-1.547269000	-3.166638000
H	-0.352511000	-0.985655000	-3.899404000
H	0.854197000	-2.281726000	-3.722640000
H	0.932543000	-0.842508000	-2.670864000
C	0.438379000	-3.480480000	-0.846276000
H	0.562147000	-3.056409000	0.164058000
H	1.419825000	-3.499727000	-1.351957000
H	0.124088000	-4.526301000	-0.709873000
C	-3.685041000	-1.561960000	1.825467000
H	-2.873756000	-1.340635000	2.535932000
H	-4.387495000	-2.247551000	2.329209000
H	-4.226151000	-0.626566000	1.619743000
C	-4.661832000	-2.769557000	-0.750700000
H	-5.285299000	-1.871804000	-0.892878000
H	-5.267302000	-3.505973000	-0.195649000
H	-4.444658000	-3.188812000	-1.745302000
C	-2.375267000	-4.058472000	0.778001000
H	-2.221695000	-4.754079000	-0.060615000
H	-3.108537000	-4.521362000	1.459622000
H	-1.423111000	-3.979570000	1.322259000
K	2.586057000	-0.954017000	0.595450000
C	2.443350000	-1.132184000	3.648889000
C	2.050117000	0.233131000	3.495862000
H	2.690390000	1.015379000	3.918332000
C	0.880246000	0.598947000	2.862401000
H	0.623404000	1.656181000	2.766784000
C	0.036236000	-0.389187000	2.211023000
H	-1.042492000	-0.129989000	2.167764000
C	0.302192000	-1.757732000	2.615683000
H	-0.420415000	-2.534561000	2.363697000
C	1.484362000	-2.100577000	3.256345000
C	3.784462000	1.019898000	-2.120796000
C	4.632409000	1.363931000	-1.051340000
C	5.547455000	0.395643000	-0.592748000
H	6.224956000	0.645894000	0.229345000
C	5.611761000	-0.874357000	-1.179344000
H	6.338025000	-1.605466000	-0.813902000
C	4.759359000	-1.201906000	-2.243773000
C	3.847538000	-0.248790000	-2.712479000
H	3.177222000	-0.492253000	-3.539590000
H	3.063627000	1.751856000	-2.493301000
C	4.552224000	2.716284000	-0.385139000
H	5.555488000	3.121366000	-0.179902000
H	3.999697000	3.440561000	-0.998142000
H	4.025081000	2.643711000	0.583928000
H	4.812528000	-2.189049000	-2.709626000
C	3.671074000	-1.503977000	4.443536000
H	3.489374000	-1.447632000	5.534960000

H	4.000022000	-2.533321000	4.228277000
H	4.519319000	-0.831367000	4.232693000
H	1.669502000	-3.154476000	3.492381000

INT1: E= -2288.986867

C	0.060896000	-2.258597000	-1.079752000
C	1.079349000	-3.304526000	-0.647573000
C	1.394849000	-4.293618000	-1.547836000
C	0.661066000	-2.928053000	-3.423872000
C	0.299350000	-1.916741000	-2.552371000
C	1.049487000	-4.205034000	-2.929200000
H	-0.973828000	-2.687137000	-0.955039000
H	1.488057000	-3.323292000	0.368298000
H	2.021088000	-5.135352000	-1.216304000
H	0.127445000	-0.904022000	-2.947563000
H	1.358922000	-4.988866000	-3.628603000
C	0.768370000	-2.657911000	-4.910266000
H	1.821564000	-2.664156000	-5.249887000
H	0.247751000	-3.441636000	-5.490372000
H	0.326171000	-1.686814000	-5.190308000
Al	0.008154000	-0.571988000	-0.137709000
O	-0.204207000	1.356025000	-0.805559000
N	-1.801906000	-0.218555000	0.431639000
N	1.690699000	0.162117000	0.421208000
C	-1.435233000	1.932405000	-0.471158000
C	-2.298648000	1.048750000	0.194533000
C	-3.554630000	1.585184000	0.543692000
H	-4.288210000	0.942621000	1.034401000
C	-3.855902000	2.933767000	0.287066000
C	-2.905404000	3.780345000	-0.309943000
H	-3.149897000	4.832382000	-0.463238000
C	-1.650247000	3.279118000	-0.701012000
C	-0.497311000	4.070932000	-1.347527000
C	0.807204000	3.541888000	-0.721451000
C	1.926192000	4.300106000	-0.330962000
H	1.940611000	5.380322000	-0.482059000
C	3.034224000	3.674066000	0.267460000
C	3.026461000	2.293075000	0.526088000
H	3.876135000	1.823596000	1.024889000
C	1.915293000	1.499586000	0.173557000
C	0.886564000	2.179664000	-0.497166000
C	-0.478406000	3.765307000	-2.868624000
H	-1.419015000	4.098019000	-3.336381000
H	0.363581000	4.286261000	-3.352028000
H	-0.365316000	2.687337000	-3.053551000
C	-0.657317000	5.581068000	-1.138763000
H	-0.678317000	5.840391000	-0.069664000
H	0.171881000	6.127119000	-1.612991000
H	-1.588243000	5.939894000	-1.602736000
C	-2.764346000	-1.252051000	0.655800000
C	-3.556202000	-1.715553000	-0.430065000
C	-4.558678000	-2.660496000	-0.172845000
H	-5.181993000	-3.014550000	-0.997681000
C	-4.755087000	-3.177526000	1.106314000
H	-5.537967000	-3.920004000	1.286059000
C	-3.930013000	-2.766192000	2.151309000
H	-4.075312000	-3.203392000	3.140136000
C	-2.925135000	-1.806481000	1.950637000
C	-2.023221000	-1.366462000	3.102453000
H	-1.006638000	-1.262355000	2.689405000
C	-1.942419000	-2.391609000	4.240386000

H	-2.902629000	-2.485081000	4.774444000
H	-1.660400000	-3.388943000	3.868667000
H	-1.191809000	-2.079379000	4.980528000
C	-2.423492000	0.012507000	3.655378000
H	-1.728628000	0.328816000	4.452275000
H	-2.412580000	0.781100000	2.871729000
H	-3.439934000	-0.023775000	4.083573000
C	-3.345698000	-1.234574000	-1.864303000
H	-2.418577000	-0.645961000	-1.892890000
C	-3.145451000	-2.400710000	-2.847013000
H	-4.060061000	-3.010053000	-2.946457000
H	-2.892711000	-2.008830000	-3.844894000
H	-2.314226000	-3.048529000	-2.533951000
C	-4.493592000	-0.318891000	-2.326832000
H	-4.605450000	0.554262000	-1.667231000
H	-4.306425000	0.047184000	-3.350256000
H	-5.452335000	-0.865830000	-2.336429000
C	2.799437000	-0.623214000	0.868604000
C	2.907368000	-0.950919000	2.242593000
C	4.005013000	-1.712969000	2.670977000
H	4.103451000	-1.976159000	3.726418000
C	4.980761000	-2.133440000	1.769241000
H	5.830943000	-2.726477000	2.118962000
C	4.865733000	-1.802962000	0.419948000
H	5.627007000	-2.146166000	-0.284274000
C	3.780699000	-1.057472000	-0.061663000
C	3.715943000	-0.708761000	-1.546682000
H	2.705432000	-0.332251000	-1.756169000
C	4.730686000	0.395307000	-1.901869000
H	5.763897000	0.050544000	-1.721673000
H	4.647964000	0.659432000	-2.969719000
H	4.569785000	1.310477000	-1.313746000
C	3.907592000	-1.931799000	-2.454680000
H	3.191152000	-2.722617000	-2.197932000
H	3.724129000	-1.644971000	-3.502958000
H	4.936038000	-2.330329000	-2.394059000
C	1.887616000	-0.435969000	3.253837000
H	0.951938000	-0.245132000	2.707643000
C	2.330716000	0.913010000	3.849755000
H	3.281945000	0.799853000	4.397932000
H	2.478891000	1.667212000	3.064177000
H	1.573399000	1.298483000	4.553942000
C	1.569982000	-1.439302000	4.369001000
H	0.765485000	-1.047472000	5.010963000
H	1.242510000	-2.406995000	3.959104000
H	2.440434000	-1.621966000	5.020270000
C	-5.223277000	3.462178000	0.657506000
H	-5.996049000	3.072542000	-0.028314000
H	-5.513051000	3.151051000	1.674681000
H	-5.262950000	4.561573000	0.613554000
C	4.259046000	4.480849000	0.634873000
H	4.640743000	4.202326000	1.630709000
H	5.078555000	4.302686000	-0.083666000
H	4.050632000	5.562049000	0.639573000

INT1-K: E= -2888.863101

K	-4.099999000	-2.195276000	-0.938907000
C	-0.652506000	-1.792645000	-1.373551000
C	-1.593033000	-1.736483000	-2.570190000
C	-2.151596000	-2.924510000	-3.003859000
C	-1.779384000	-3.975391000	-0.824003000

C	-1.229372000	-2.790469000	-0.358000000
C	-2.184030000	-4.094482000	-2.189108000
H	0.319703000	-2.216758000	-1.726491000
H	-1.598827000	-0.862533000	-3.228967000
H	-2.654504000	-2.950268000	-3.981542000
H	-0.952326000	-2.730941000	0.701994000
H	-2.604451000	-5.030455000	-2.566097000
C	-2.011854000	-5.131769000	0.125824000
H	-3.008983000	-5.592229000	0.000962000
H	-1.274422000	-5.931076000	-0.063523000
H	-1.897097000	-4.825292000	1.178254000
Al	0.011963000	-0.211295000	-0.434574000
O	0.965170000	1.547394000	-0.813697000
N	-1.306162000	1.003744000	0.265095000
N	1.777502000	-0.691298000	0.127505000
C	0.309764000	2.658210000	-0.258177000
C	-0.907921000	2.339018000	0.350876000
C	-1.567854000	3.404140000	0.989193000
H	-2.526293000	3.223709000	1.479178000
C	-1.000745000	4.690090000	1.011653000
C	0.247563000	4.926875000	0.410723000
H	0.681925000	5.925639000	0.465159000
C	0.941213000	3.890939000	-0.236500000
C	2.330392000	3.980162000	-0.896322000
C	3.072418000	2.692163000	-0.496088000
C	4.413955000	2.584202000	-0.092749000
H	5.059703000	3.462758000	-0.091825000
C	4.934360000	1.348852000	0.332917000
C	4.109264000	0.214375000	0.408039000
H	4.506403000	-0.726947000	0.791528000
C	2.753515000	0.284480000	0.032062000
C	2.335874000	1.520807000	-0.476844000
C	2.149822000	4.004758000	-2.436962000
H	1.582226000	4.898568000	-2.740647000
H	3.132308000	4.023625000	-2.934103000
H	1.606073000	3.116234000	-2.789371000
C	3.091337000	5.236380000	-0.458883000
H	3.239250000	5.260900000	0.630843000
H	4.077483000	5.277932000	-0.943950000
H	2.546536000	6.143976000	-0.758064000
C	-2.702134000	0.777244000	0.444359000
C	-3.633791000	1.298512000	-0.502465000
C	-5.008906000	1.160537000	-0.247176000
H	-5.727578000	1.580095000	-0.955588000
C	-5.475974000	0.501037000	0.890785000
H	-6.549565000	0.419057000	1.080956000
C	-4.557680000	-0.050387000	1.789462000
H	-4.929979000	-0.571498000	2.672330000
C	-3.172805000	0.075126000	1.589302000
C	-2.189775000	-0.511335000	2.594760000
H	-1.350009000	-0.919125000	2.013440000
C	-2.765051000	-1.677404000	3.406463000
H	-3.559975000	-1.350871000	4.096603000
H	-3.176227000	-2.461290000	2.750128000
H	-1.976748000	-2.135875000	4.017843000
C	-1.617907000	0.577769000	3.519398000
H	-0.856032000	0.152259000	4.192069000
H	-1.148892000	1.388135000	2.944593000
H	-2.415926000	1.016224000	4.141279000
C	-3.203102000	1.986452000	-1.797700000
H	-2.109795000	1.908555000	-1.870700000

C	-3.789241000	1.281786000	-3.034658000
H	-4.888174000	1.365180000	-3.069662000
H	-3.398350000	1.742432000	-3.956287000
H	-3.512087000	0.216834000	-3.053439000
C	-3.570685000	3.481320000	-1.809433000
H	-3.124637000	4.012967000	-0.957988000
H	-3.208398000	3.957173000	-2.734966000
H	-4.663881000	3.620241000	-1.769103000
C	2.213085000	-2.038095000	0.348694000
C	2.035393000	-2.628481000	1.626146000
C	2.470027000	-3.945734000	1.827472000
H	2.340967000	-4.418518000	2.801836000
C	3.076252000	-4.669288000	0.801761000
H	3.412104000	-5.694513000	0.978378000
C	3.245443000	-4.082728000	-0.449169000
H	3.715326000	-4.657783000	-1.250470000
C	2.817892000	-2.772153000	-0.705987000
C	3.034896000	-2.193476000	-2.103305000
H	2.505209000	-1.231461000	-2.154038000
C	4.525576000	-1.915617000	-2.373522000
H	5.109674000	-2.850487000	-2.343914000
H	4.660628000	-1.467600000	-3.371948000
H	4.950032000	-1.224643000	-1.631759000
C	2.453478000	-3.095144000	-3.207577000
H	1.396275000	-3.335004000	-3.019968000
H	2.522810000	-2.590454000	-4.185196000
H	3.008441000	-4.043704000	-3.289907000
C	1.425639000	-1.829698000	2.774728000
H	0.645232000	-1.184172000	2.343483000
C	2.461707000	-0.894069000	3.423946000
H	3.288965000	-1.479372000	3.859055000
H	2.890141000	-0.193568000	2.695422000
H	1.999227000	-0.303646000	4.232667000
C	0.758695000	-2.702268000	3.844210000
H	0.234083000	-2.068244000	4.575614000
H	0.031730000	-3.401996000	3.403768000
H	1.497760000	-3.292216000	4.409645000
C	-1.757035000	5.816626000	1.676168000
H	-2.605090000	6.144149000	1.049454000
H	-2.176350000	5.502734000	2.645503000
H	-1.115432000	6.693338000	1.849829000
C	6.389169000	1.224814000	0.721454000
H	6.507492000	0.649605000	1.653731000
H	6.959395000	0.691986000	-0.059300000
H	6.860549000	2.208752000	0.864351000

INT1-K-para: E= -2888.863558

K	-4.638222000	-0.541834000	-0.342400000
C	-1.585853000	-1.094466000	-1.900438000
C	-2.468048000	0.051473000	-2.396898000
C	-3.601831000	-0.226368000	-3.138085000
C	-3.639845000	-2.488905000	-2.312811000
C	-2.514609000	-2.253446000	-1.536331000
C	-4.195603000	-1.522347000	-3.195606000
H	-0.898985000	-1.424794000	-2.738905000
H	-2.098836000	1.082777000	-2.358512000
H	-4.119439000	0.605144000	-3.640156000
H	-2.180206000	-3.040975000	-0.856895000
Al	-0.260813000	-0.422062000	-0.630040000
O	0.948511000	0.943683000	-1.412465000
N	-0.728607000	0.967912000	0.598943000

N	1.348766000	-1.397880000	-0.382691000
C	1.013764000	2.082633000	-0.579535000
C	0.147385000	2.051396000	0.523816000
C	0.311669000	3.099870000	1.445043000
H	-0.306238000	3.114556000	2.345087000
C	1.267752000	4.106890000	1.230977000
C	2.106157000	4.067028000	0.101738000
H	2.857248000	4.846979000	-0.028753000
C	2.001218000	3.021206000	-0.829589000
C	2.898167000	2.771904000	-2.064499000
C	3.263637000	1.274854000	-1.995384000
C	4.551567000	0.719868000	-2.074711000
H	5.398923000	1.335290000	-2.378204000
C	4.772793000	-0.621969000	-1.712288000
C	3.725192000	-1.398472000	-1.190673000
H	3.929042000	-2.392308000	-0.787896000
C	2.424802000	-0.869297000	-1.081910000
C	2.243661000	0.409080000	-1.635596000
C	2.084142000	3.060641000	-3.352379000
H	1.775428000	4.117543000	-3.378054000
H	2.699787000	2.853055000	-4.241496000
H	1.181609000	2.436322000	-3.408900000
C	4.144251000	3.662554000	-2.048238000
H	4.751711000	3.496101000	-1.146573000
H	4.770863000	3.463632000	-2.930047000
H	3.858385000	4.724262000	-2.084552000
C	-1.916283000	1.074063000	1.368764000
C	-2.867529000	2.119233000	1.127603000
C	-4.040030000	2.159205000	1.899386000
H	-4.757314000	2.966498000	1.730603000
C	-4.327219000	1.185303000	2.859538000
H	-5.249669000	1.238399000	3.443817000
C	-3.411461000	0.155262000	3.072194000
H	-3.625612000	-0.605642000	3.826650000
C	-2.201346000	0.089060000	2.361563000
C	-1.240537000	-1.051003000	2.679222000
H	-0.303280000	-0.855264000	2.142577000
C	-1.781383000	-2.403102000	2.186654000
H	-2.713464000	-2.670330000	2.713062000
H	-2.007247000	-2.386986000	1.109056000
H	-1.045051000	-3.200706000	2.364264000
C	-0.883384000	-1.123146000	4.172712000
H	-0.075181000	-1.855312000	4.324440000
H	-0.539256000	-0.150051000	4.553799000
H	-1.744644000	-1.440583000	4.783116000
C	-2.707840000	3.177021000	0.031396000
H	-1.790506000	2.955325000	-0.527604000
C	-3.865062000	3.132317000	-0.983970000
H	-4.833146000	3.375221000	-0.515441000
H	-3.696488000	3.870092000	-1.784995000
H	-3.934150000	2.144965000	-1.463735000
C	-2.578146000	4.597223000	0.611756000
H	-1.750452000	4.672290000	1.329351000
H	-2.390700000	5.323954000	-0.195046000
H	-3.504439000	4.902528000	1.125974000
C	1.618711000	-2.302523000	0.689067000
C	2.270090000	-1.829302000	1.861612000
C	2.446124000	-2.720158000	2.930302000
H	2.937096000	-2.369875000	3.842063000
C	2.005793000	-4.040913000	2.854238000
H	2.150586000	-4.717100000	3.700923000

C	1.383782000	-4.497954000	1.692155000
H	1.049384000	-5.535447000	1.639766000
C	1.179752000	-3.647101000	0.596589000
C	0.538869000	-4.158100000	-0.688743000
H	-0.013789000	-3.320495000	-1.134059000
C	1.618730000	-4.561145000	-1.709279000
H	2.242195000	-5.381885000	-1.316089000
H	1.155538000	-4.902152000	-2.649653000
H	2.278201000	-3.714103000	-1.947234000
C	-0.465357000	-5.296569000	-0.474119000
H	-1.200808000	-5.043549000	0.305763000
H	-1.016243000	-5.489058000	-1.407569000
H	0.028406000	-6.238206000	-0.182217000
C	2.809131000	-0.402794000	1.991660000
H	2.575042000	0.142674000	1.070361000
C	4.342110000	-0.405819000	2.133214000
H	4.656774000	-0.868325000	3.083262000
H	4.815871000	-0.959234000	1.309817000
H	4.730712000	0.625399000	2.116340000
C	2.160364000	0.391541000	3.136449000
H	2.618431000	1.390945000	3.207857000
H	1.087519000	0.539686000	2.960030000
H	2.284931000	-0.109753000	4.109730000
C	1.377007000	5.239455000	2.224724000
H	2.312203000	5.805201000	2.099458000
H	0.540239000	5.950021000	2.104747000
H	1.338109000	4.868781000	3.261547000
C	6.151094000	-1.226388000	-1.837659000
H	6.227643000	-1.842961000	-2.750029000
H	6.932298000	-0.453304000	-1.895093000
H	6.381242000	-1.883929000	-0.984115000
C	-5.378144000	-1.823468000	-4.082175000
H	-6.017800000	-2.619415000	-3.657246000
H	-6.018303000	-0.933624000	-4.232122000
H	-5.094523000	-2.169073000	-5.096676000
H	-4.181292000	-3.437174000	-2.172278000

INT1-D: E= -2350.825928

K	3.391846000	1.862739000	-1.226504000
Al	-0.311764000	-0.064209000	0.099652000
N	-1.925873000	-0.599765000	0.749231000
N	0.895643000	-1.224337000	0.822648000
C	-0.892990000	-2.312806000	2.147995000
C	-1.983800000	-1.619364000	1.709389000
C	-3.139494000	-0.105416000	0.161028000
C	0.512626000	-2.241028000	1.737951000
C	2.274848000	-1.166126000	0.457656000
C	-3.728660000	1.090734000	0.632209000
C	-3.713025000	-0.816005000	-0.925278000
C	-3.347194000	-1.947499000	2.266486000
C	-0.076065000	1.493160000	-1.041670000
C	0.655833000	1.294812000	-2.369976000
C	1.129192000	2.413387000	-3.027641000
C	1.397412000	-3.150953000	2.234686000
C	-3.137990000	1.879976000	1.795013000
C	3.219405000	-0.587785000	1.347698000
C	2.694329000	-1.688157000	-0.799056000
C	-5.455737000	0.875323000	-1.073008000
C	1.100646000	3.728374000	-0.969504000
C	0.633039000	2.619586000	-0.275965000
C	-4.886608000	1.563292000	-0.004351000

C	1.290751000	3.672008000	-2.381250000
C	-4.870280000	-0.307332000	-1.527343000
C	2.797523000	0.027237000	2.677983000
C	-4.131284000	2.013239000	2.962087000
C	4.060482000	-1.643988000	-1.124278000
C	3.024644000	1.549824000	2.679366000
C	-2.635898000	3.261145000	1.339114000
C	4.575638000	-0.569500000	0.978675000
C	-3.044072000	-2.075418000	-1.470105000
C	4.999685000	-1.100369000	-0.242140000
C	1.689426000	-2.305708000	-1.767755000
C	-2.134937000	-1.719827000	-2.661076000
C	2.092674000	-2.189568000	-3.242838000
C	3.495187000	-0.642679000	3.872534000
C	1.383457000	-3.767919000	-1.396026000
C	-4.036521000	-3.183598000	-1.848430000
H	-1.086359000	-3.069866000	2.908859000
H	-3.827271000	-1.050214000	2.687411000
H	-3.281219000	-2.713963000	3.049503000
H	-4.021611000	-2.312594000	1.475125000
H	-1.139597000	1.796615000	-1.253368000
H	0.588013000	0.345287000	-2.908538000
H	-2.271171000	1.314160000	2.167657000
H	-6.356815000	1.261288000	-1.556701000
H	0.530125000	2.694716000	0.813588000
H	-5.348912000	2.490068000	0.344440000
H	-5.319159000	-0.836989000	-2.370371000
H	1.719358000	-0.146479000	2.791078000
H	-5.016010000	2.604181000	2.673962000
H	-3.657262000	2.522303000	3.817174000
H	-4.486948000	1.029634000	3.306707000
H	4.401431000	-2.051319000	-2.077477000
H	4.094724000	1.798637000	2.571637000
H	2.679417000	1.993838000	3.627156000
H	2.468450000	2.037886000	1.862040000
H	-1.893853000	3.179281000	0.531521000
H	-2.164222000	3.798707000	2.178437000
H	-3.467589000	3.883643000	0.970145000
H	5.312104000	-0.141958000	1.664367000
H	-2.400589000	-2.483017000	-0.677188000
H	6.061342000	-1.098131000	-0.504592000
H	0.746723000	-1.747058000	-1.656022000
H	-2.729825000	-1.345234000	-3.509985000
H	-1.560687000	-2.597473000	-3.000988000
H	-1.419269000	-0.921996000	-2.405074000
H	2.361510000	-1.155944000	-3.510229000
H	1.250800000	-2.494639000	-3.883792000
H	2.942181000	-2.846399000	-3.491818000
H	3.305278000	-1.725725000	3.875949000
H	3.113652000	-0.226601000	4.818970000
H	4.585550000	-0.477961000	3.855072000
H	2.286758000	-4.389490000	-1.514689000
H	0.600515000	-4.175577000	-2.056611000
H	1.045986000	-3.857681000	-0.354595000
H	-4.705347000	-3.425758000	-1.007445000
H	-3.494317000	-4.101253000	-2.128205000
H	-4.665598000	-2.903001000	-2.708709000
H	1.053129000	-3.915976000	2.930882000
H	2.447731000	-3.166915000	1.947611000
H	1.644009000	4.547030000	-2.932255000
C	1.476674000	4.989324000	-0.219781000

H	0.715329000	5.771141000	-0.386195000
H	1.538750000	4.818635000	0.866958000
H	2.437299000	5.413343000	-0.563124000
H	1.469438000	2.307943000	-4.067720000

INT1-D-para: E= -2350.821124

K	-3.308339000	2.226917000	0.267485000
Al	0.336230000	-0.187031000	0.256845000
N	1.959770000	-1.003080000	0.192197000
N	-0.877095000	-1.541132000	0.131539000
C	0.949709000	-3.218636000	0.222766000
C	2.042966000	-2.399907000	0.202225000
C	3.118925000	-0.202047000	-0.067032000
C	-0.481572000	-2.906019000	0.158219000
C	-2.266702000	-1.272445000	-0.063522000
C	3.330748000	0.297024000	-1.379265000
C	3.993765000	0.142547000	0.989902000
C	3.426000000	-3.005891000	0.190889000
C	0.156208000	1.749628000	0.308781000
C	-0.571458000	2.300443000	1.538583000
C	-1.005901000	3.616560000	1.517023000
C	-1.383011000	-3.927006000	0.102251000
C	2.323591000	0.019794000	-2.494298000
C	-2.747319000	-0.980741000	-1.372648000
C	-3.160064000	-1.303437000	1.039825000
C	5.373346000	1.366135000	-0.597674000
C	-0.978733000	3.637079000	-0.894529000
C	-0.540361000	2.324006000	-0.929173000
C	4.474008000	1.070207000	-1.622211000
C	-1.153886000	4.367137000	0.316541000
C	5.123373000	0.919575000	0.698654000
C	-1.810721000	-0.970523000	-2.576531000
C	2.535804000	-1.358674000	-3.141542000
C	-4.523915000	-1.045566000	0.816023000
C	-2.169526000	0.084906000	-3.631383000
C	2.249849000	1.126359000	-3.552943000
C	-4.118927000	-0.731954000	-1.546196000
C	3.665256000	-0.235603000	2.429965000
C	-5.006049000	-0.767907000	-0.465405000
C	-2.676002000	-1.602850000	2.454008000
C	2.947280000	0.937690000	3.123928000
C	-2.840280000	-0.374887000	3.366578000
C	-1.710872000	-2.370940000	-3.209946000
C	-3.367677000	-2.838997000	3.051481000
C	4.885629000	-0.694590000	3.240099000
H	1.158382000	-4.288440000	0.258043000
H	3.997626000	-2.667636000	-0.688267000
H	3.374834000	-4.102302000	0.173218000
H	4.008919000	-2.696590000	1.072430000
H	1.229680000	2.089311000	0.321334000
H	-0.517891000	1.775203000	2.498335000
H	-1.334581000	4.075860000	2.461565000
H	1.323672000	-0.008675000	-2.024817000
H	6.260761000	1.968875000	-0.807545000
H	-1.289154000	4.111363000	-1.837951000
H	-0.474742000	1.810756000	-1.893769000
H	4.664516000	1.458869000	-2.623838000
H	5.812756000	1.192876000	1.500473000
H	-0.806161000	-0.723706000	-2.202580000
H	3.538792000	-1.420873000	-3.594852000
H	1.790361000	-1.533623000	-3.935030000

H	2.436032000	-2.170262000	-2.407765000
H	-5.221142000	-1.079470000	1.657203000
H	-3.093281000	-0.169864000	-4.176183000
H	-1.364111000	0.153625000	-4.379739000
H	-2.295116000	1.081447000	-3.180254000
H	2.108599000	2.114533000	-3.089567000
H	1.398049000	0.941333000	-4.227033000
H	3.156371000	1.160790000	-4.178693000
H	-4.505171000	-0.518573000	-2.544750000
H	2.955920000	-1.074783000	2.400734000
H	-6.073618000	-0.592935000	-0.625942000
H	-1.603549000	-1.830689000	2.388330000
H	3.605052000	1.820734000	3.176903000
H	2.654385000	0.667408000	4.152128000
H	2.040382000	1.242500000	2.578262000
H	-2.304145000	0.499264000	2.962812000
H	-2.439249000	-0.579972000	4.372408000
H	-3.903049000	-0.102129000	3.485827000
H	-1.404785000	-3.122504000	-2.469042000
H	-0.975806000	-2.368450000	-4.031615000
H	-2.686146000	-2.675875000	-3.625551000
H	-4.450517000	-2.677920000	3.184826000
H	-2.943859000	-3.070250000	4.042106000
H	-3.222727000	-3.717492000	2.406309000
H	5.424992000	-1.509804000	2.731734000
H	4.571321000	-1.059203000	4.231313000
H	5.601687000	0.125712000	3.409538000
H	-1.032914000	-4.959432000	0.112783000
H	-2.455610000	-3.759624000	0.017696000
C	-1.552285000	5.822255000	0.323787000
H	-2.153930000	6.079097000	1.215349000
H	-0.687546000	6.515165000	0.328217000
H	-2.152737000	6.089490000	-0.565826000

TS2: E= -2288.985137

Al	-0.584560000	0.180489000	-0.703126000
O	1.289673000	-0.313530000	-1.393567000
N	0.388507000	1.517065000	0.250195000
N	-0.505594000	-1.638219000	-0.113515000
C	2.271338000	0.416025000	-0.701774000
C	1.764943000	1.381800000	0.188362000
C	2.743945000	2.078257000	0.925412000
H	2.422231000	2.845103000	1.633043000
C	4.109109000	1.785239000	0.776124000
C	4.536337000	0.774083000	-0.104561000
H	5.600782000	0.548165000	-0.182219000
C	3.597735000	0.055510000	-0.865435000
C	3.882419000	-1.105705000	-1.846670000
C	2.805535000	-2.171517000	-1.555178000
C	3.017157000	-3.545282000	-1.336121000
H	3.994022000	-3.991221000	-1.527758000
C	1.983155000	-4.348249000	-0.819679000
C	0.755465000	-3.778125000	-0.446037000
H	0.002986000	-4.377511000	0.071033000
C	0.516254000	-2.401175000	-0.632899000
C	1.526150000	-1.698858000	-1.319778000
C	3.725586000	-0.586219000	-3.299109000
H	4.456144000	0.214186000	-3.498530000
H	3.894461000	-1.406009000	-4.015847000
H	2.717861000	-0.182984000	-3.471062000
C	5.297186000	-1.665051000	-1.664496000

H	5.456343000	-2.036156000	-0.641222000
H	5.478517000	-2.492827000	-2.366573000
H	6.048745000	-0.888878000	-1.873609000
C	-0.160266000	2.753737000	0.702439000
C	0.175011000	3.963687000	0.026623000
C	-0.337515000	5.173533000	0.509700000
H	-0.079005000	6.103802000	-0.003482000
C	-1.203750000	5.213735000	1.601706000
H	-1.600165000	6.168591000	1.958377000
C	-1.588056000	4.020951000	2.206736000
H	-2.302906000	4.043730000	3.033270000
C	-1.088799000	2.782607000	1.775822000
C	-1.624247000	1.517720000	2.435850000
H	-1.090033000	0.657634000	2.008944000
C	-3.120193000	1.329199000	2.121062000
H	-3.725949000	2.102318000	2.624446000
H	-3.314641000	1.398768000	1.040107000
H	-3.461602000	0.341737000	2.470045000
C	-1.379155000	1.486780000	3.953247000
H	-1.672052000	0.505560000	4.360741000
H	-0.319466000	1.655530000	4.197704000
H	-1.970917000	2.259284000	4.472549000
C	0.990720000	3.988215000	-1.266949000
H	1.315477000	2.965079000	-1.489676000
C	0.123550000	4.439214000	-2.457124000
H	-0.227060000	5.476954000	-2.327485000
H	0.708080000	4.397699000	-3.392219000
H	-0.761450000	3.794526000	-2.573698000
C	2.259360000	4.849114000	-1.147193000
H	2.898675000	4.506893000	-0.319646000
H	2.850608000	4.796050000	-2.076746000
H	2.012462000	5.909884000	-0.972504000
C	-1.127993000	-2.064848000	1.099756000
C	-0.371197000	-2.070713000	2.305848000
C	-1.024648000	-2.387528000	3.506545000
H	-0.457058000	-2.380463000	4.441280000
C	-2.383688000	-2.696866000	3.531872000
H	-2.876801000	-2.930138000	4.480115000
C	-3.110189000	-2.709243000	2.340907000
H	-4.172426000	-2.963327000	2.361109000
C	-2.505497000	-2.399321000	1.113234000
C	-3.307429000	-2.485165000	-0.176466000
H	-2.762931000	-1.939364000	-0.955906000
C	-3.403689000	-3.947353000	-0.647978000
H	-3.948303000	-4.570432000	0.083423000
H	-3.937352000	-4.003088000	-1.611591000
H	-2.403348000	-4.387979000	-0.788602000
C	-4.693875000	-1.836996000	-0.085293000
H	-4.616998000	-0.797540000	0.264495000
H	-5.151960000	-1.799087000	-1.084626000
H	-5.374653000	-2.391411000	0.584879000
C	1.125297000	-1.753173000	2.345595000
H	1.456787000	-1.514960000	1.329439000
C	1.940227000	-2.975963000	2.802571000
H	1.712727000	-3.241935000	3.848696000
H	1.726001000	-3.851310000	2.172037000
H	3.020821000	-2.766012000	2.734934000
C	1.460046000	-0.521052000	3.199735000
H	2.547894000	-0.343272000	3.197846000
H	0.981711000	0.377943000	2.792003000
H	1.134360000	-0.642938000	4.245657000

C	-2.775027000	-0.204041000	-2.648399000
H	-2.200348000	-1.040495000	-3.064302000
C	-2.173867000	0.683758000	-1.611311000
C	-2.753351000	2.056224000	-1.658632000
H	-2.216689000	2.901446000	-1.220895000
C	-4.049991000	2.218584000	-2.106598000
H	-4.509074000	3.214613000	-2.033076000
C	-4.784484000	1.171463000	-2.715495000
H	-5.803686000	1.338362000	-3.078492000
C	-4.075814000	-0.003036000	-3.081189000
H	-2.485397000	0.227306000	-0.472360000
C	5.120170000	2.582603000	1.568602000
H	4.809250000	2.693926000	2.620211000
H	6.114706000	2.110489000	1.555876000
H	5.229407000	3.601704000	1.156956000
C	2.198651000	-5.831300000	-0.623646000
H	1.700281000	-6.412748000	-1.419265000
H	3.267715000	-6.094965000	-0.641711000
H	1.778196000	-6.174653000	0.335811000
C	-4.726050000	-0.994018000	-4.023967000
H	-4.060003000	-1.842582000	-4.252155000
H	-5.651717000	-1.408611000	-3.582736000
H	-5.021224000	-0.521874000	-4.979255000

TS2-K: E= -2888.849471

K	-2.024351000	-4.085712000	0.099870000
C	-0.197582000	-1.944872000	-1.193541000
C	-1.361638000	-2.289523000	-2.038089000
C	-1.418277000	-3.470951000	-2.753483000
C	0.556230000	-4.317369000	-1.614862000
C	0.622631000	-3.135737000	-0.873710000
C	-0.461577000	-4.506691000	-2.582766000
H	0.493266000	-1.032807000	-1.995295000
H	-2.115849000	-1.526319000	-2.221114000
H	-2.258408000	-3.629010000	-3.442317000
H	1.390565000	-3.064317000	-0.100117000
H	-0.513684000	-5.425358000	-3.171469000
C	1.572817000	-5.408776000	-1.367368000
H	1.134278000	-6.417152000	-1.455574000
H	2.383171000	-5.342629000	-2.114548000
H	2.043288000	-5.311305000	-0.376089000
Al	0.016826000	-0.207902000	-0.404986000
O	0.173584000	1.794698000	-0.849908000
N	-1.721229000	0.429615000	0.165695000
N	1.815110000	0.107360000	0.189121000
C	-0.886258000	2.570307000	-0.371063000
C	-1.905065000	1.807766000	0.213353000
C	-2.939873000	2.538012000	0.822442000
H	-3.752683000	2.002185000	1.316376000
C	-2.927381000	3.944756000	0.814221000
C	-1.865557000	4.643046000	0.215952000
H	-1.868003000	5.733316000	0.238106000
C	-0.798758000	3.951570000	-0.385375000
C	0.450008000	4.568833000	-1.043354000
C	1.643759000	3.696607000	-0.613659000
C	2.926358000	4.147038000	-0.256534000
H	3.169397000	5.208934000	-0.304247000
C	3.907382000	3.236223000	0.172588000
C	3.602542000	1.871792000	0.310393000
H	4.354399000	1.181427000	0.695948000
C	2.317396000	1.384476000	-0.001282000

C	1.433129000	2.330721000	-0.536857000
C	0.292242000	4.480011000	-2.584308000
H	-0.584916000	5.059571000	-2.913262000
H	1.189000000	4.881772000	-3.081666000
H	0.159054000	3.438134000	-2.910341000
C	0.641599000	6.035251000	-0.640039000
H	0.758944000	6.143622000	0.448425000
H	1.531538000	6.457858000	-1.128827000
H	-0.218903000	6.641150000	-0.960330000
C	-2.853463000	-0.389202000	0.439687000
C	-3.959434000	-0.430065000	-0.462668000
C	-5.063958000	-1.235731000	-0.145762000
H	-5.915810000	-1.262364000	-0.828604000
C	-5.101349000	-2.005496000	1.014084000
H	-5.978575000	-2.615708000	1.245331000
C	-4.012798000	-1.977235000	1.888425000
H	-4.060852000	-2.565487000	2.806114000
C	-2.884677000	-1.180489000	1.624753000
C	-1.721260000	-1.148899000	2.614107000
H	-0.794048000	-1.206099000	2.018629000
C	-1.698393000	-2.333454000	3.588921000
H	-2.551791000	-2.309905000	4.285261000
H	-1.716882000	-3.310411000	3.075308000
H	-0.785105000	-2.304946000	4.195392000
C	-1.676444000	0.172983000	3.401257000
H	-0.801237000	0.194046000	4.070400000
H	-1.613969000	1.041556000	2.734038000
H	-2.582927000	0.281007000	4.019372000
C	-4.002764000	0.360678000	-1.772114000
H	-2.985281000	0.730106000	-1.968458000
C	-4.432905000	-0.504179000	-2.974548000
H	-5.506464000	-0.748819000	-2.931832000
H	-4.265540000	0.048727000	-3.912445000
H	-3.876104000	-1.449349000	-3.033564000
C	-4.943363000	1.579265000	-1.683840000
H	-4.637226000	2.282343000	-0.900327000
H	-4.949934000	2.123679000	-2.642079000
H	-5.976878000	1.255511000	-1.475177000
C	2.774101000	-0.919741000	0.464549000
C	2.888828000	-1.441850000	1.779280000
C	3.801580000	-2.480756000	2.013979000
H	3.899286000	-2.903862000	3.014496000
C	4.605433000	-2.981453000	0.990202000
H	5.311550000	-3.790889000	1.193781000
C	4.509696000	-2.438739000	-0.288223000
H	5.145604000	-2.829221000	-1.086340000
C	3.602957000	-1.409857000	-0.578563000
C	3.547275000	-0.865585000	-2.003824000
H	2.735534000	-0.128336000	-2.054561000
C	4.854866000	-0.144269000	-2.377096000
H	5.711636000	-0.838779000	-2.369357000
H	4.781622000	0.283755000	-3.390437000
H	5.074851000	0.676305000	-1.678229000
C	3.212866000	-1.962579000	-3.028863000
H	2.268232000	-2.465229000	-2.778883000
H	3.113404000	-1.523761000	-4.034985000
H	4.006560000	-2.726709000	-3.078654000
C	2.068794000	-0.856305000	2.926114000
H	1.074446000	-0.616098000	2.521455000
C	2.668776000	0.467570000	3.435023000
H	3.685615000	0.302997000	3.829177000

H	2.731254000	1.220640000	2.639835000
H	2.051501000	0.884016000	4.248757000
C	1.878412000	-1.821990000	4.102188000
H	1.179092000	-1.392217000	4.836556000
H	1.484959000	-2.797120000	3.775499000
H	2.824698000	-2.003887000	4.636647000
C	-4.078755000	4.691273000	1.446732000
H	-3.864925000	5.765850000	1.546236000
H	-4.996148000	4.588019000	0.841110000
H	-4.310786000	4.295912000	2.449104000
C	5.304570000	3.712650000	0.495553000
H	6.011095000	3.425893000	-0.302821000
H	5.349772000	4.807090000	0.601174000
H	5.677091000	3.263294000	1.430212000

TS2-K-para: E= -2888.845321

K	-3.801898000	-2.448199000	0.406480000
C	-1.166032000	-1.641158000	-1.041679000
C	-2.380258000	-1.350091000	-1.833174000
C	-3.080363000	-2.336663000	-2.505294000
C	-1.819533000	-4.040839000	-1.378871000
C	-1.089697000	-3.076546000	-0.685432000
C	-2.815007000	-3.724152000	-2.332176000
H	-0.133447000	-1.255194000	-1.900379000
H	-2.643325000	-0.309310000	-2.012911000
H	-3.910129000	-2.036212000	-3.160232000
H	-0.376370000	-3.410058000	0.071673000
Al	-0.060099000	-0.231331000	-0.349298000
O	1.069184000	1.392104000	-0.920808000
N	-1.206843000	1.228750000	0.213779000
N	1.670783000	-0.845688000	0.201553000
C	0.573983000	2.619089000	-0.468564000
C	-0.660383000	2.507940000	0.183087000
C	-1.147546000	3.688074000	0.770717000
H	-2.092076000	3.662961000	1.317447000
C	-0.422641000	4.890081000	0.677021000
C	0.815282000	4.923730000	0.014272000
H	1.368894000	5.862263000	-0.029128000
C	1.350851000	3.760510000	-0.566959000
C	2.706173000	3.626176000	-1.286433000
C	3.310652000	2.287666000	-0.824234000
C	4.658120000	2.039724000	-0.511087000
H	5.403835000	2.826901000	-0.627366000
C	5.058208000	0.778689000	-0.036034000
C	4.109281000	-0.232050000	0.190323000
H	4.422309000	-1.187982000	0.613020000
C	2.742952000	-0.012665000	-0.076000000
C	2.439241000	1.224554000	-0.660299000
C	2.450924000	3.562188000	-2.815597000
H	1.973301000	4.492210000	-3.162710000
H	3.402796000	3.430784000	-3.353784000
H	1.792781000	2.719769000	-3.073904000
C	3.634201000	4.806874000	-0.980305000
H	3.845071000	4.887270000	0.096355000
H	4.589287000	4.696305000	-1.514166000
H	3.183612000	5.751439000	-1.319372000
C	-2.582627000	1.118010000	0.564125000
C	-3.600673000	1.613400000	-0.306506000
C	-4.943637000	1.496886000	0.084121000
H	-5.724203000	1.881557000	-0.575776000
C	-5.308045000	0.902623000	1.289849000

H	-6.360456000	0.836495000	1.578585000
C	-4.313085000	0.406700000	2.135055000
H	-4.605800000	-0.036629000	3.088136000
C	-2.951879000	0.502053000	1.795525000
C	-1.885717000	-0.028971000	2.751606000
H	-1.150650000	-0.576405000	2.137381000
C	-2.420082000	-1.019957000	3.794036000
H	-3.103247000	-0.535398000	4.509631000
H	-2.962054000	-1.867677000	3.339989000
H	-1.589870000	-1.441251000	4.373393000
C	-1.132374000	1.112759000	3.457324000
H	-0.333889000	0.709580000	4.100698000
H	-0.671879000	1.801591000	2.738253000
H	-1.823121000	1.691913000	4.092056000
C	-3.303991000	2.269557000	-1.656935000
H	-2.249593000	2.069440000	-1.898047000
C	-4.167497000	1.697854000	-2.799791000
H	-5.217417000	2.020988000	-2.713143000
H	-3.796196000	2.066122000	-3.769252000
H	-4.156071000	0.599662000	-2.827975000
C	-3.500091000	3.797920000	-1.611327000
H	-2.849509000	4.274568000	-0.868892000
H	-3.270996000	4.240260000	-2.594408000
H	-4.546407000	4.046548000	-1.366935000
C	1.987029000	-2.205853000	0.520729000
C	1.888500000	-2.656637000	1.862866000
C	2.168582000	-4.002081000	2.143830000
H	2.091907000	-4.371963000	3.166970000
C	2.561235000	-4.883796000	1.137414000
H	2.776314000	-5.928575000	1.377168000
C	2.686110000	-4.424138000	-0.170534000
H	3.001248000	-5.116443000	-0.954978000
C	2.406421000	-3.092022000	-0.506439000
C	2.574962000	-2.653310000	-1.959003000
H	2.243433000	-1.609788000	-2.039478000
C	4.052145000	-2.706348000	-2.389243000
H	4.441405000	-3.737887000	-2.358478000
H	4.164387000	-2.338616000	-3.422552000
H	4.683926000	-2.084035000	-1.738404000
C	1.695121000	-3.471514000	-2.918612000
H	0.637310000	-3.426287000	-2.625387000
H	1.786148000	-3.077673000	-3.944018000
H	1.998252000	-4.531699000	-2.943571000
C	1.538193000	-1.688374000	2.990352000
H	0.785798000	-0.989840000	2.595388000
C	2.753337000	-0.838484000	3.405902000
H	3.561285000	-1.484197000	3.789018000
H	3.153277000	-0.257813000	2.565649000
H	2.474939000	-0.131276000	4.205306000
C	0.943179000	-2.373117000	4.227279000
H	0.595602000	-1.616522000	4.948292000
H	0.095146000	-3.026380000	3.969707000
H	1.692732000	-2.987436000	4.751468000
C	-1.002305000	6.144125000	1.288882000
H	-0.267014000	6.961763000	1.321788000
H	-1.871836000	6.500231000	0.709060000
H	-1.355925000	5.962187000	2.316867000
C	6.515715000	0.493173000	0.242635000
H	6.949259000	-0.143566000	-0.548257000
H	7.112187000	1.416814000	0.289006000
H	6.644884000	-0.045742000	1.195058000

C	-3.544750000	-4.781870000	-3.121192000
H	-3.069428000	-4.995077000	-4.098540000
H	-3.581232000	-5.742586000	-2.577911000
H	-4.586486000	-4.486442000	-3.342569000
H	-1.649503000	-5.097133000	-1.129319000

TS2-D: E= -2350.803457

K	3.365991000	2.319211000	-0.520889000
Al	-0.253760000	-0.104906000	0.007431000
N	-1.861616000	-0.598841000	0.733065000
N	0.897695000	-1.410516000	0.600214000
C	-0.846988000	-2.427527000	2.010459000
C	-1.908946000	-1.635784000	1.677643000
C	-3.098459000	-0.185569000	0.126920000
C	0.523835000	-2.438783000	1.495874000
C	2.287277000	-1.230623000	0.342453000
C	-3.699297000	1.046557000	0.469636000
C	-3.703708000	-1.038287000	-0.836165000
C	-3.243324000	-1.887585000	2.340733000
C	0.406313000	1.437897000	-0.887819000
C	1.153831000	1.412682000	-2.160511000
C	1.446813000	2.581844000	-2.840109000
C	1.394261000	-3.399090000	1.919806000
C	-3.058237000	2.019077000	1.452187000
C	3.117096000	-0.658007000	1.345400000
C	2.830900000	-1.578994000	-0.924667000
C	-5.533231000	0.550846000	-1.057560000
C	0.690376000	3.939439000	-0.967834000
C	0.410734000	2.770072000	-0.255774000
C	-4.920944000	1.389982000	-0.131512000
C	1.205403000	3.864426000	-2.283798000
C	-4.919364000	-0.652310000	-1.411054000
C	2.556128000	-0.231302000	2.699430000
C	-3.874963000	2.145377000	2.749471000
C	4.195826000	-1.345834000	-1.160570000
C	2.542578000	1.301581000	2.840380000
C	-2.829042000	3.397761000	0.809492000
C	4.479653000	-0.456886000	1.068223000
C	-2.999682000	-2.314615000	-1.293878000
C	5.021481000	-0.797979000	-0.174953000
C	1.951573000	-2.221443000	-1.995297000
C	-2.103329000	-2.013983000	-2.510428000
C	2.479488000	-2.054220000	-3.425562000
C	3.294579000	-0.898470000	3.869384000
C	1.698826000	-3.709282000	-1.687982000
C	-3.953869000	-3.478367000	-1.593937000
H	-1.036854000	-3.182987000	2.774185000
H	-3.660063000	-0.954417000	2.750191000
H	-3.148905000	-2.622018000	3.151443000
H	-3.984932000	-2.262553000	1.617455000
H	-0.950224000	1.039122000	-1.293481000
H	1.358027000	0.457256000	-2.649171000
H	-2.074600000	1.608314000	1.725916000
H	-6.485153000	0.835912000	-1.513211000
H	0.070912000	2.871922000	0.780822000
H	-5.398771000	2.336709000	0.132484000
H	-5.395223000	-1.295894000	-2.153746000
H	1.513083000	-0.566405000	2.747205000
H	-4.882575000	2.544294000	2.547166000
H	-3.379703000	2.829601000	3.458390000
H	-3.997019000	1.171068000	3.247181000

H	4.630219000	-1.609646000	-2.125811000
H	3.564477000	1.721055000	2.807739000
H	2.101824000	1.602612000	3.804393000
H	1.937577000	1.772899000	2.046381000
H	-2.264411000	3.309322000	-0.129913000
H	-2.258842000	4.052613000	1.488972000
H	-3.784371000	3.902064000	0.589973000
H	5.129035000	-0.037669000	1.842334000
H	-2.340650000	-2.649711000	-0.481049000
H	6.087651000	-0.652474000	-0.370839000
H	0.969472000	-1.723241000	-1.953015000
H	-2.713587000	-1.742555000	-3.387565000
H	-1.482427000	-2.886264000	-2.773500000
H	-1.436247000	-1.156543000	-2.317644000
H	2.720791000	-1.005473000	-3.659192000
H	1.719509000	-2.395198000	-4.145621000
H	3.384479000	-2.658696000	-3.600796000
H	3.266554000	-1.993039000	3.764165000
H	2.814239000	-0.635058000	4.825627000
H	4.348736000	-0.579813000	3.929922000
H	2.645424000	-4.274016000	-1.725829000
H	1.014087000	-4.142698000	-2.435555000
H	1.260358000	-3.849795000	-0.691336000
H	-4.623602000	-3.677462000	-0.742283000
H	-3.380258000	-4.396717000	-1.798424000
H	-4.581852000	-3.284968000	-2.478801000
H	1.064611000	-4.159252000	2.628551000
H	2.424324000	-3.447734000	1.567546000
H	1.429392000	4.771856000	-2.849091000
C	0.470537000	5.286648000	-0.318818000
H	-0.529173000	5.675432000	-0.580013000
H	0.513327000	5.223169000	0.780520000
H	1.207075000	6.035083000	-0.654525000
H	1.921997000	2.510772000	-3.826395000

TS2-D-para: E= -2350.800276

K	-3.483473000	2.164153000	0.156292000
Al	0.297631000	-0.080461000	0.083993000
N	1.949832000	-0.662736000	0.620124000
N	-0.777220000	-1.549624000	0.360617000
C	1.054964000	-2.789431000	1.444567000
C	2.071617000	-1.892102000	1.286395000
C	3.148602000	-0.066721000	0.093832000
C	-0.330077000	-2.743447000	0.973053000
C	-2.182227000	-1.393104000	0.181677000
C	3.735992000	-0.631081000	-1.071243000
C	3.731323000	1.059913000	0.715640000
C	3.437044000	-2.231042000	1.838714000
C	-0.461151000	1.591631000	-0.418284000
C	-0.547464000	2.756812000	0.481264000
C	-0.930470000	4.013656000	0.014700000
C	-1.143843000	-3.819303000	1.174011000
C	3.052555000	-1.782403000	-1.806932000
C	-2.753145000	-1.473606000	-1.118415000
C	-3.004118000	-1.120524000	1.309888000
C	5.505419000	1.035716000	-0.955060000
C	-1.623331000	3.058243000	-2.076074000
C	-1.245397000	1.794008000	-1.653062000
C	4.913341000	-0.066586000	-1.574400000
C	-1.453953000	4.224349000	-1.280220000
C	4.912391000	1.592124000	0.174129000

C	-1.882941000	-1.804154000	-2.328589000
C	4.027167000	-2.791696000	-2.428518000
C	-4.384618000	-0.942965000	1.119377000
C	-2.458478000	-1.323516000	-3.666441000
C	2.089089000	-1.228596000	-2.874497000
C	-4.136650000	-1.275637000	-1.261295000
C	3.108714000	1.726150000	1.935831000
C	-4.953154000	-1.020231000	-0.156078000
C	-2.416558000	-0.988173000	2.712288000
C	2.748216000	3.193021000	1.645444000
C	-2.464641000	0.470353000	3.202136000
C	-1.566787000	-3.310231000	-2.389838000
C	-3.083133000	-1.942781000	3.713899000
C	4.010630000	1.614514000	3.176666000
H	1.300432000	-3.695198000	2.000793000
H	4.167680000	-2.391866000	1.029838000
H	3.398799000	-3.139044000	2.454823000
H	3.831573000	-1.402676000	2.447294000
H	0.910064000	1.388796000	-0.908175000
H	-0.203171000	2.670679000	1.516840000
H	-0.879452000	4.865395000	0.706155000
H	2.447021000	-2.339750000	-1.078885000
H	6.426179000	1.463612000	-1.360238000
H	-2.118793000	3.154802000	-3.051363000
H	-1.418242000	0.951583000	-2.326697000
H	5.377036000	-0.485786000	-2.469781000
H	5.374861000	2.461501000	0.648415000
H	-0.918741000	-1.291062000	-2.185134000
H	4.602721000	-2.356447000	-3.261379000
H	3.472746000	-3.653330000	-2.833981000
H	4.744717000	-3.168438000	-1.682495000
H	-5.026912000	-0.754437000	1.984603000
H	-3.345694000	-1.906349000	-3.963869000
H	-1.708124000	-1.450111000	-4.462256000
H	-2.745011000	-0.260551000	-3.637642000
H	1.405605000	-0.472467000	-2.451910000
H	1.483638000	-2.034379000	-3.321287000
H	2.648677000	-0.725012000	-3.679874000
H	-4.591948000	-1.337482000	-2.250767000
H	2.175272000	1.188569000	2.161531000
H	-6.032165000	-0.898432000	-0.287209000
H	-1.359169000	-1.272994000	2.656738000
H	3.652421000	3.803120000	1.485077000
H	2.198513000	3.633396000	2.494053000
H	2.116916000	3.278665000	0.749267000
H	-1.911167000	1.141654000	2.522658000
H	-2.002623000	0.565041000	4.198066000
H	-3.504463000	0.833530000	3.292815000
H	-1.090859000	-3.661575000	-1.464954000
H	-0.890931000	-3.524229000	-3.234246000
H	-2.492050000	-3.891752000	-2.538634000
H	-4.146971000	-1.700149000	3.874573000
H	-2.579695000	-1.883034000	4.692419000
H	-3.011882000	-2.980853000	3.356927000
H	4.236231000	0.564818000	3.420739000
H	3.524212000	2.070710000	4.054845000
H	4.970709000	2.133151000	3.019997000
H	-0.756941000	-4.709384000	1.670913000
H	-2.182356000	-3.834737000	0.844502000
C	-1.811031000	5.596979000	-1.793503000
H	-0.983944000	6.070431000	-2.356522000

H	-2.676270000	5.567639000	-2.479762000
H	-2.063324000	6.287217000	-0.969850000

2: E= -2289.091284

Al	0.032033000	-0.893580000	-0.357528000
O	-0.026491000	1.322395000	-0.047770000
N	-1.729044000	-0.499133000	0.497474000
N	1.872922000	-0.352690000	0.181329000
C	-1.143985000	1.789990000	0.610326000
C	-2.083408000	0.772832000	0.877263000
C	-3.253868000	1.203727000	1.539336000
H	-4.033343000	0.474099000	1.768306000
C	-3.414483000	2.545382000	1.930879000
C	-2.410202000	3.492346000	1.677045000
H	-2.551739000	4.523065000	2.005858000
C	-1.233297000	3.114090000	0.998370000
C	-0.068022000	4.043360000	0.602828000
C	1.236706000	3.227245000	0.689588000
C	2.500179000	3.710642000	1.089599000
H	2.626998000	4.755415000	1.377083000
C	3.607293000	2.848960000	1.121658000
C	3.469770000	1.486913000	0.796058000
H	4.330648000	0.820235000	0.877109000
C	2.217715000	0.952984000	0.416446000
C	1.171476000	1.895411000	0.325463000
C	-0.274561000	4.479305000	-0.872288000
H	-1.220798000	5.034071000	-0.981249000
H	0.557664000	5.121196000	-1.204508000
H	-0.314496000	3.602151000	-1.534473000
C	-0.014902000	5.292734000	1.491843000
H	0.127114000	5.025547000	2.549763000
H	0.809942000	5.953845000	1.186342000
H	-0.944465000	5.874420000	1.401215000
C	-2.770845000	-1.467342000	0.450501000
C	-3.751895000	-1.396013000	-0.577309000
C	-4.792155000	-2.336209000	-0.592128000
H	-5.551719000	-2.280179000	-1.377064000
C	-4.868809000	-3.344185000	0.365152000
H	-5.685112000	-4.072231000	0.337641000
C	-3.888508000	-3.427740000	1.354564000
H	-3.953930000	-4.229520000	2.091895000
C	-2.835003000	-2.504482000	1.421069000
C	-1.788465000	-2.589992000	2.528857000
H	-0.821591000	-2.358327000	2.057631000
C	-1.666295000	-3.987214000	3.148898000
H	-2.571806000	-4.270374000	3.712293000
H	-1.487937000	-4.755358000	2.379913000
H	-0.825829000	-4.014790000	3.857742000
C	-2.035479000	-1.540530000	3.626913000
H	-1.235424000	-1.580534000	4.386065000
H	-2.060937000	-0.522769000	3.216114000
H	-2.997134000	-1.726687000	4.136499000
C	-3.706954000	-0.331463000	-1.671379000
H	-2.776876000	0.235641000	-1.546308000
C	-3.654610000	-0.953133000	-3.076061000
H	-4.583629000	-1.497309000	-3.318272000
H	-3.516538000	-0.166672000	-3.836013000
H	-2.808652000	-1.648007000	-3.168009000
C	-4.877235000	0.660459000	-1.554671000
H	-4.882019000	1.156963000	-0.573371000
H	-4.798447000	1.441462000	-2.330016000

H	-5.847805000	0.152091000	-1.689084000
C	2.910793000	-1.317228000	0.290784000
C	3.121710000	-1.986947000	1.523851000
C	4.177047000	-2.903292000	1.628065000
H	4.352606000	-3.417663000	2.576388000
C	5.013067000	-3.167948000	0.542849000
H	5.835716000	-3.882325000	0.643364000
C	4.787929000	-2.522561000	-0.672033000
H	5.438335000	-2.738001000	-1.524410000
C	3.741076000	-1.601352000	-0.823635000
C	3.524877000	-0.913565000	-2.166945000
H	2.561207000	-0.390520000	-2.112702000
C	4.610500000	0.141384000	-2.442326000
H	5.610091000	-0.324084000	-2.502863000
H	4.417971000	0.654891000	-3.399714000
H	4.634969000	0.902052000	-1.647950000
C	3.427724000	-1.917431000	-3.325770000
H	2.671388000	-2.687321000	-3.116456000
H	3.126534000	-1.402622000	-4.252330000
H	4.391359000	-2.421281000	-3.516114000
C	2.237920000	-1.689778000	2.728693000
H	1.318483000	-1.227651000	2.341722000
C	2.900262000	-0.667562000	3.669853000
H	3.843187000	-1.069674000	4.080498000
H	3.130599000	0.268579000	3.141320000
H	2.234529000	-0.427776000	4.516619000
C	1.828330000	-2.953234000	3.495097000
H	1.095622000	-2.700019000	4.278257000
H	1.368959000	-3.691590000	2.821462000
H	2.687061000	-3.431125000	3.996516000
C	-0.337708000	0.527500000	-3.024871000
H	-0.394340000	1.450424000	-2.438324000
C	-0.143370000	-0.695086000	-2.350596000
C	-0.076214000	-1.845676000	-3.163543000
H	0.069338000	-2.824264000	-2.692185000
C	-0.189250000	-1.776437000	-4.557293000
H	-0.123410000	-2.688164000	-5.160675000
C	-0.385480000	-0.543481000	-5.184691000
H	-0.477328000	-0.489390000	-6.275121000
C	-0.468849000	0.630305000	-4.418949000
H	0.146136000	-2.471894000	-0.023677000
C	-4.692706000	2.951357000	2.630541000
H	-4.889339000	2.314084000	3.509554000
H	-4.657973000	3.997478000	2.972778000
H	-5.564601000	2.846382000	1.960966000
C	4.972014000	3.369424000	1.515327000
H	5.670368000	3.347007000	0.660077000
H	4.924761000	4.407506000	1.880020000
H	5.423538000	2.751448000	2.310013000
C	-0.731605000	1.965713000	-5.076335000
H	-0.378888000	2.799797000	-4.449054000
H	-0.237088000	2.043748000	-6.058938000
H	-1.812941000	2.121561000	-5.245087000

2-K: E= -2888.933301

K	-3.245315000	-2.999577000	-0.656050000
C	-0.370514000	-2.171352000	-0.792634000
C	-0.531571000	-2.920960000	-1.982004000
C	-0.594824000	-4.323588000	-1.979213000
C	-0.277289000	-4.336461000	0.433315000
C	-0.261908000	-2.928440000	0.392240000

C	-0.467118000	-5.031384000	-0.775006000
H	-0.200188000	0.008274000	-2.572445000
H	-0.575163000	-2.390083000	-2.940052000
H	-0.709007000	-4.872025000	-2.919817000
H	-0.104236000	-2.407251000	1.338024000
H	-0.477702000	-6.125732000	-0.777553000
C	-0.011568000	-5.061230000	1.729808000
H	-0.269423000	-6.129746000	1.670179000
H	1.059845000	-4.983579000	1.981583000
H	-0.568038000	-4.615385000	2.569604000
Al	-0.011284000	-0.154704000	-0.975413000
O	0.517633000	1.907076000	-0.848601000
N	-1.571608000	0.692711000	-0.031512000
N	1.854979000	-0.188699000	-0.333810000
C	-0.416269000	2.749148000	-0.288028000
C	-1.580343000	2.076135000	0.118672000
C	-2.591825000	2.898054000	0.653590000
H	-3.539868000	2.458088000	0.969778000
C	-2.379767000	4.279838000	0.833140000
C	-1.153949000	4.864603000	0.482211000
H	-1.003896000	5.930786000	0.656846000
C	-0.130957000	4.089220000	-0.098001000
C	1.245264000	4.594496000	-0.578649000
C	2.272861000	3.467349000	-0.349544000
C	3.628900000	3.623451000	-0.000301000
H	4.051440000	4.618751000	0.143016000
C	4.453082000	2.498367000	0.161360000
C	3.930227000	1.197980000	0.027540000
H	4.574407000	0.334693000	0.205087000
C	2.569195000	0.994471000	-0.277324000
C	1.835636000	2.167987000	-0.521305000
C	1.152742000	4.864987000	-2.103937000
H	0.402788000	5.645306000	-2.311339000
H	2.128534000	5.196807000	-2.492968000
H	0.860193000	3.952795000	-2.644809000
C	1.653768000	5.885593000	0.142217000
H	1.727757000	5.732703000	1.229350000
H	2.626231000	6.245521000	-0.224527000
H	0.924589000	6.686904000	-0.048499000
C	-2.827922000	0.078508000	0.162804000
C	-3.810015000	0.135283000	-0.870110000
C	-5.085545000	-0.408622000	-0.640237000
H	-5.844093000	-0.346454000	-1.425310000
C	-5.407692000	-1.007453000	0.580675000
H	-6.413007000	-1.399965000	0.756546000
C	-4.435419000	-1.085701000	1.583751000
H	-4.696460000	-1.547925000	2.537539000
C	-3.145202000	-0.557114000	1.399070000
C	-2.118287000	-0.623931000	2.522803000
H	-1.128725000	-0.628002000	2.044017000
C	-2.246745000	-1.889875000	3.380760000
H	-3.175629000	-1.897055000	3.973668000
H	-2.227181000	-2.805733000	2.767425000
H	-1.412786000	-1.950778000	4.093290000
C	-2.174953000	0.634168000	3.408660000
H	-1.397115000	0.585544000	4.188016000
H	-2.011650000	1.547277000	2.821132000
H	-3.154893000	0.717467000	3.908512000
C	-3.501817000	0.771362000	-2.220347000
H	-2.481718000	1.170181000	-2.172762000
C	-3.504805000	-0.280912000	-3.341753000

H	-4.486890000	-0.776355000	-3.441499000
H	-3.268807000	0.185994000	-4.311019000
H	-2.728305000	-1.043200000	-3.163650000
C	-4.445393000	1.943763000	-2.530482000
H	-4.405550000	2.696241000	-1.728283000
H	-4.147157000	2.434326000	-3.470768000
H	-5.492359000	1.615334000	-2.644826000
C	2.549045000	-1.366625000	0.069592000
C	2.692803000	-1.671995000	1.450003000
C	3.393656000	-2.830707000	1.817203000
H	3.523071000	-3.069630000	2.875513000
C	3.925576000	-3.688898000	0.856593000
H	4.468420000	-4.587667000	1.162144000
C	3.755754000	-3.398565000	-0.496953000
H	4.165853000	-4.082824000	-1.241944000
C	3.076338000	-2.245328000	-0.913278000
C	2.928071000	-1.927378000	-2.397860000
H	1.990036000	-1.365535000	-2.517135000
C	4.065065000	-1.009901000	-2.884343000
H	5.041813000	-1.511382000	-2.774141000
H	3.930290000	-0.755862000	-3.948997000
H	4.099494000	-0.072125000	-2.313472000
C	2.827775000	-3.174262000	-3.286698000
H	2.068692000	-3.876272000	-2.910398000
H	2.548155000	-2.883161000	-4.312247000
H	3.787514000	-3.713113000	-3.356197000
C	2.083719000	-0.787214000	2.536363000
H	1.318330000	-0.164467000	2.047076000
C	3.115713000	0.161063000	3.175831000
H	3.932018000	-0.417193000	3.641220000
H	3.555288000	0.842441000	2.437007000
H	2.642424000	0.772012000	3.962617000
C	1.394963000	-1.603205000	3.643389000
H	0.809330000	-0.938055000	4.298358000
H	0.719480000	-2.367394000	3.233661000
H	2.127172000	-2.121834000	4.282988000
C	-3.495506000	5.121950000	1.409113000
H	-3.152847000	6.136957000	1.660508000
H	-4.330568000	5.220588000	0.693552000
H	-3.910998000	4.668685000	2.324583000
C	5.916091000	2.661671000	0.506027000
H	6.561343000	2.253913000	-0.290738000
H	6.187518000	3.718346000	0.649838000
H	6.171009000	2.118198000	1.431763000

2-K-para: E= -2888.930820

K	-3.744910000	2.324693000	-0.475676000
C	-2.202558000	-0.249348000	-0.607741000
C	-3.011236000	-0.309825000	-1.765558000
C	-4.386584000	-0.597686000	-1.725579000
C	-4.253011000	-0.805216000	0.664091000
C	-2.884528000	-0.510103000	0.602969000
C	-5.035333000	-0.858034000	-0.505585000
H	-0.160454000	0.166179000	-2.495904000
H	-2.540403000	-0.151531000	-2.742979000
H	-4.958430000	-0.651805000	-2.658492000
H	-2.327758000	-0.513754000	1.541127000
Al	-0.174329000	-0.053874000	-0.893597000
O	1.963016000	-0.003502000	-0.899738000
N	0.276746000	1.686801000	0.003942000
N	0.339280000	-1.847876000	-0.268523000

C	2.551282000	1.128018000	-0.381850000
C	1.612819000	2.070768000	0.069969000
C	2.159121000	3.273618000	0.558553000
H	1.496724000	4.070822000	0.902248000
C	3.554790000	3.446425000	0.653900000
C	4.428568000	2.420721000	0.264169000
H	5.503879000	2.567691000	0.373241000
C	3.927136000	1.218524000	-0.272553000
C	4.757151000	0.025062000	-0.789981000
C	3.964958000	-1.267060000	-0.502068000
C	4.500985000	-2.526948000	-0.169303000
H	5.579990000	-2.664296000	-0.088089000
C	3.651024000	-3.621803000	0.055023000
C	2.252135000	-3.469736000	0.002080000
H	1.606064000	-4.320132000	0.228129000
C	1.672117000	-2.217812000	-0.284794000
C	2.588250000	-1.198528000	-0.595574000
C	4.904371000	0.172736000	-2.327777000
H	5.434762000	1.106367000	-2.575415000
H	5.469500000	-0.677932000	-2.741007000
H	3.917651000	0.197261000	-2.813602000
C	6.149838000	-0.013936000	-0.147997000
H	6.085108000	-0.120540000	0.945232000
H	6.736388000	-0.855402000	-0.544780000
H	6.712375000	0.903447000	-0.376479000
C	-0.641037000	2.736288000	0.225402000
C	-0.887623000	3.689630000	-0.806962000
C	-1.741418000	4.776732000	-0.551776000
H	-1.913256000	5.518912000	-1.336191000
C	-2.357982000	4.936436000	0.692086000
H	-2.997327000	5.802118000	0.885815000
C	-2.136133000	3.986189000	1.694955000
H	-2.614616000	4.122288000	2.666435000
C	-1.289073000	2.882962000	1.487121000
C	-1.027745000	1.889417000	2.612884000
H	-0.810527000	0.922826000	2.136217000
C	-2.229341000	1.706711000	3.549713000
H	-2.440310000	2.615733000	4.136082000
H	-3.144660000	1.435943000	2.997710000
H	-2.027051000	0.902991000	4.270974000
C	0.227120000	2.277146000	3.416710000
H	0.424692000	1.526246000	4.198821000
H	1.114141000	2.339569000	2.772776000
H	0.089971000	3.254867000	3.908841000
C	-0.246130000	3.552149000	-2.183020000
H	0.407367000	2.672099000	-2.158232000
C	-1.306395000	3.277209000	-3.262774000
H	-2.044564000	4.095681000	-3.335138000
H	-0.832803000	3.169442000	-4.251338000
H	-1.831826000	2.328732000	-3.061852000
C	0.624673000	4.769079000	-2.532568000
H	1.388781000	4.935899000	-1.758167000
H	1.143834000	4.603744000	-3.490194000
H	0.027174000	5.690909000	-2.633844000
C	-0.582940000	-2.824083000	0.209231000
C	-0.749622000	-3.020866000	1.606280000
C	-1.645265000	-4.005462000	2.049104000
H	-1.773889000	-4.175553000	3.120881000
C	-2.381885000	-4.769615000	1.146486000
H	-3.075465000	-5.532946000	1.509992000
C	-2.235300000	-4.551524000	-0.223288000

H	-2.824326000	-5.149437000	-0.921450000
C	-1.342776000	-3.588829000	-0.714670000
C	-1.167182000	-3.390354000	-2.216847000
H	-0.884510000	-2.339277000	-2.375334000
C	-0.010233000	-4.251060000	-2.757205000
H	-0.225347000	-5.323735000	-2.613241000
H	0.135043000	-4.074072000	-3.836045000
H	0.934907000	-4.023386000	-2.245727000
C	-2.447011000	-3.644078000	-3.024741000
H	-3.301702000	-3.089408000	-2.609071000
H	-2.303214000	-3.321152000	-4.068679000
H	-2.714799000	-4.713525000	-3.053289000
C	0.007339000	-2.177898000	2.630780000
H	0.386847000	-1.291561000	2.098204000
C	1.220832000	-2.919700000	3.221573000
H	0.895705000	-3.838858000	3.737999000
H	1.941868000	-3.199228000	2.443550000
H	1.742778000	-2.284454000	3.956757000
C	-0.896516000	-1.690779000	3.775251000
H	-0.370377000	-0.937178000	4.383181000
H	-1.828626000	-1.242312000	3.402600000
H	-1.176038000	-2.513592000	4.452829000
C	4.092604000	4.752611000	1.193114000
H	5.187762000	4.731733000	1.297299000
H	3.836105000	5.595110000	0.527717000
H	3.663679000	4.985173000	2.182448000
C	4.222646000	-4.984112000	0.376009000
H	3.982524000	-5.713189000	-0.416796000
H	5.317469000	-4.954074000	0.483141000
H	3.801591000	-5.383442000	1.314261000
C	-6.491021000	-1.253760000	-0.454188000
H	-6.590994000	-2.353143000	-0.449400000
H	-6.985127000	-0.882986000	0.457958000
H	-7.049135000	-0.880604000	-1.326992000
H	-4.717185000	-1.024223000	1.631507000

2-D: E= -2350.900398

K	2.377786000	0.109534000	-2.605444000
Al	-0.138719000	-0.023109000	-0.251169000
N	-1.671354000	-0.479708000	0.704792000
N	1.222527000	-0.591271000	0.914171000
C	-0.388795000	-1.041173000	2.715404000
C	-1.567545000	-0.867513000	2.038563000
C	-2.940036000	-0.535576000	0.044777000
C	0.995351000	-0.992058000	2.241207000
C	2.519983000	-0.801410000	0.388263000
C	-3.774073000	0.612102000	-0.025119000
C	-3.350844000	-1.747077000	-0.573969000
C	-2.862409000	-1.108647000	2.781586000
C	0.007776000	1.911638000	-0.755794000
C	0.044857000	2.339953000	-2.100893000
C	0.190984000	3.689826000	-2.447983000
C	2.007009000	-1.368533000	3.082347000
C	-3.382488000	1.942051000	0.610666000
C	3.487966000	0.243892000	0.386855000
C	2.853410000	-2.067376000	-0.183080000
C	-5.412897000	-0.670713000	-1.296309000
C	0.227837000	4.285584000	-0.091557000
C	0.093286000	2.923467000	0.219286000
C	-5.000971000	0.518528000	-0.699003000
C	0.289066000	4.656983000	-1.444107000

C	-4.586920000	-1.791710000	-1.233444000
C	3.213320000	1.580575000	1.062531000
C	-4.354264000	2.360212000	1.728248000
C	4.117902000	-2.244870000	-0.768460000
C	3.440933000	2.776754000	0.124527000
C	-3.268906000	3.060073000	-0.439593000
C	4.741509000	0.019796000	-0.213872000
C	-2.477904000	-2.998564000	-0.547316000
C	5.059748000	-1.210630000	-0.793297000
C	1.877860000	-3.237117000	-0.137874000
C	-2.041248000	-3.399701000	-1.966475000
C	1.615211000	-3.839255000	-1.526678000
C	4.044325000	1.725543000	2.349710000
C	2.364074000	-4.309551000	0.853027000
C	-3.167812000	-4.171669000	0.168948000
H	-0.482445000	-1.316040000	3.766726000
H	-3.506753000	-0.217044000	2.761671000
H	-2.673462000	-1.382522000	3.827625000
H	-3.446438000	-1.914769000	2.310530000
H	0.120963000	-0.779301000	-1.690110000
H	-0.100541000	1.606764000	-2.908737000
H	-2.390805000	1.806000000	1.062956000
H	-6.375173000	-0.723428000	-1.812762000
H	0.053020000	2.646234000	1.279788000
H	-5.648730000	1.397490000	-0.753940000
H	-4.909777000	-2.722491000	-1.707549000
H	2.155351000	1.573428000	1.351305000
H	-5.367288000	2.542423000	1.333106000
H	-4.011966000	3.292345000	2.207598000
H	-4.437100000	1.590070000	2.509760000
H	4.377810000	-3.216216000	-1.197372000
H	4.494476000	2.859169000	-0.191827000
H	3.176322000	3.716932000	0.632333000
H	2.803960000	2.722894000	-0.773094000
H	-2.578685000	2.782197000	-1.247724000
H	-2.886182000	3.985373000	0.020566000
H	-4.249773000	3.289121000	-0.888601000
H	5.488832000	0.817616000	-0.204647000
H	-1.569316000	-2.758375000	0.022354000
H	6.044727000	-1.372196000	-1.239997000
H	0.923900000	-2.847396000	0.241585000
H	-2.909502000	-3.643886000	-2.600365000
H	-1.389927000	-4.287761000	-1.937400000
H	-1.483979000	-2.582072000	-2.448136000
H	1.180218000	-3.092539000	-2.210321000
H	0.893936000	-4.667847000	-1.452747000
H	2.532551000	-4.246892000	-1.983476000
H	3.827348000	0.902641000	3.045601000
H	3.802723000	2.674286000	2.856833000
H	5.126317000	1.726551000	2.133204000
H	3.318831000	-4.755193000	0.524979000
H	1.622898000	-5.121598000	0.933460000
H	2.504298000	-3.872015000	1.852170000
H	-3.461731000	-3.900206000	1.194386000
H	-2.490494000	-5.039527000	0.230757000
H	-4.076231000	-4.495331000	-0.365492000
H	1.780387000	-1.694865000	4.097778000
H	3.051791000	-1.375671000	2.772164000
H	0.401475000	5.711900000	-1.711594000
C	0.289315000	5.315310000	1.009820000
H	-0.697047000	5.432770000	1.491117000

H	0.995454000	5.012948000	1.800321000
H	0.597066000	6.303066000	0.634714000
H	0.208243000	3.994229000	-3.499251000

2-D-para: E= -2350.899395

K	-2.391428000	0.352497000	-2.483358000
Al	0.141680000	-0.008572000	-0.178403000
N	1.689310000	-0.540936000	0.713998000
N	-1.201259000	-0.749234000	0.908367000
C	0.424577000	-1.332117000	2.657525000
C	1.597362000	-1.058772000	2.003752000
C	2.953541000	-0.527056000	0.042686000
C	-0.960688000	-1.281062000	2.185994000
C	-2.498248000	-0.923652000	0.367478000
C	3.370386000	-1.677943000	-0.679180000
C	3.777545000	0.629471000	0.064356000
C	2.899449000	-1.328771000	2.723901000
C	-0.047628000	1.969608000	-0.438667000
C	-0.131964000	2.867886000	0.648254000
C	-0.280908000	4.243163000	0.471600000
C	-1.958718000	-1.777361000	2.980130000
C	2.508270000	-2.934856000	-0.752550000
C	-2.811006000	-2.120137000	-0.347096000
C	-3.487628000	0.093947000	0.494570000
C	5.416278000	-0.525358000	-1.324755000
C	-0.264652000	3.941663000	-1.910743000
C	-0.103775000	2.560719000	-1.716514000
C	4.600960000	-1.655628000	-1.349446000
C	-0.360746000	4.807895000	-0.814511000
C	4.999118000	0.604872000	-0.625428000
C	-1.812829000	-3.266704000	-0.444324000
C	3.209214000	-4.155862000	-0.134160000
C	-4.740431000	-0.083623000	-0.122978000
C	-1.571256000	-3.717620000	-1.892688000
C	2.070570000	-3.222097000	-2.198455000
C	-4.076190000	-2.253767000	-0.942737000
C	3.381455000	1.896833000	0.814104000
C	-5.038258000	-1.243427000	-0.841746000
C	-3.238373000	1.348240000	1.322116000
C	3.241854000	3.097534000	-0.137154000
C	-3.495305000	2.640094000	0.529761000
C	-2.255090000	-4.445626000	0.440680000
C	-4.066944000	1.323114000	2.618632000
C	4.365109000	2.231398000	1.949331000
H	0.526275000	-1.698973000	3.679670000
H	3.508608000	-2.067801000	2.180151000
H	2.719798000	-1.706442000	3.738783000
H	3.514566000	-0.418761000	2.790020000
H	-0.103133000	-0.590552000	-1.699639000
H	-0.074794000	2.480176000	1.671362000
H	-0.338715000	4.899942000	1.346033000
H	1.600040000	-2.748822000	-0.162721000
H	6.374282000	-0.525038000	-1.851747000
H	-0.291872000	4.353389000	-2.925239000
H	0.038090000	1.932301000	-2.609323000
H	4.927547000	-2.540321000	-1.902588000
H	5.638690000	1.491276000	-0.610119000
H	-0.858146000	-2.897388000	-0.047027000
H	4.121726000	-4.425940000	-0.691181000
H	2.540519000	-5.032532000	-0.144932000
H	3.498762000	-3.966108000	0.910702000

H	-5.503579000	0.691876000	-0.016966000
H	-2.489582000	-4.099096000	-2.369458000
H	-0.830868000	-4.531809000	-1.919439000
H	-1.169928000	-2.895021000	-2.506465000
H	1.500689000	-2.374436000	-2.608379000
H	1.431515000	-4.118318000	-2.241459000
H	2.938731000	-3.400812000	-2.853918000
H	-4.320485000	-3.173052000	-1.481233000
H	2.397253000	1.712815000	1.265968000
H	-6.022861000	-1.371430000	-1.300024000
H	-2.179583000	1.331508000	1.607310000
H	4.212933000	3.370419000	-0.583072000
H	2.860353000	3.977480000	0.405132000
H	2.539077000	2.882118000	-0.953410000
H	-2.854881000	2.707014000	-0.364821000
H	-3.254549000	3.520530000	1.144825000
H	-4.550001000	2.731811000	0.220194000
H	-2.379893000	-4.115911000	1.482257000
H	-1.496962000	-5.245596000	0.420455000
H	-3.208911000	-4.874627000	0.088533000
H	-5.149708000	1.323850000	2.406079000
H	-3.844170000	2.211808000	3.231753000
H	-3.827617000	0.429944000	3.213153000
H	4.470972000	1.397699000	2.659702000
H	4.018361000	3.113751000	2.512271000
H	5.369222000	2.461662000	1.556411000
H	-1.720345000	-2.200791000	3.956255000
H	-3.002975000	-1.787072000	2.668157000
C	-0.518664000	6.298397000	-0.988940000
H	0.350998000	6.839080000	-0.578412000
H	-0.616465000	6.578527000	-2.048683000
H	-1.408380000	6.672491000	-0.455214000
